# Fluorogenic Granzyme A Substrates Enable Real‐Time Imaging of Adaptive Immune Cell Activity

**DOI:** 10.1002/ange.202216142

**Published:** 2023-01-16

**Authors:** Zhiming Cheng, Emily J Thompson, Lorena Mendive‐Tapia, Jamie I Scott, Sam Benson, Takanori Kitamura, Ana Senan‐Salinas, Youhani Samarakoon, Edward W Roberts, Maykel A Arias, Julian Pardo, Eva M Galvez, Marc Vendrell

**Affiliations:** ^1^ Centre for Inflammation Research The University of Edinburgh Edinburgh UK; ^2^ MRC Centre for Reproductive Health The University of Edinburgh Edinburgh UK; ^3^ Instituto de Carboquimica CSIC Zaragoza Spain; ^4^ Cancer Research UK Beatson Institute Glasgow UK; ^5^ CIBERINFEC Instituto de Salud Carlos III Zaragoza Spain; ^6^ Aragón Health Research Institute Biomedical Research Centre of Aragón and Dpt of Microbiology Preventive Medicine and Public Health Zaragoza Spain

**Keywords:** Cancer, Fluorescence, Hemicyanine, Peptides, Probes

## Abstract

Cytotoxic immune cells, including T lymphocytes (CTLs) and natural killer (NK) cells, are essential components of the host response against tumors. CTLs and NK cells secrete granzyme A (GzmA) upon recognition of cancer cells; however, there are very few tools that can detect physiological levels of active GzmA with high spatiotemporal resolution. Herein, we report the rational design of the near‐infrared fluorogenic substrates for human GzmA and mouse GzmA. These activity‐based probes display very high catalytic efficiency and selectivity over other granzymes, as shown in tissue lysates from wild‐type and GzmA knock‐out mice. Furthermore, we demonstrate that the probes can image how adaptive immune cells respond to antigen‐driven recognition of cancer cells in real time.

## Graphical abstract

We describe NIR fluorogenic substrates for granzyme A with rapid responses and enzyme sensitivity at physiological levels. We demonstrate that these probes can be used for real‐time imaging of CTLs and NK cells in co‐cultures with cancer cells and in ex vivo tissues, opening new avenues to monitor the functions of the immune system in the tumor microenvironment.

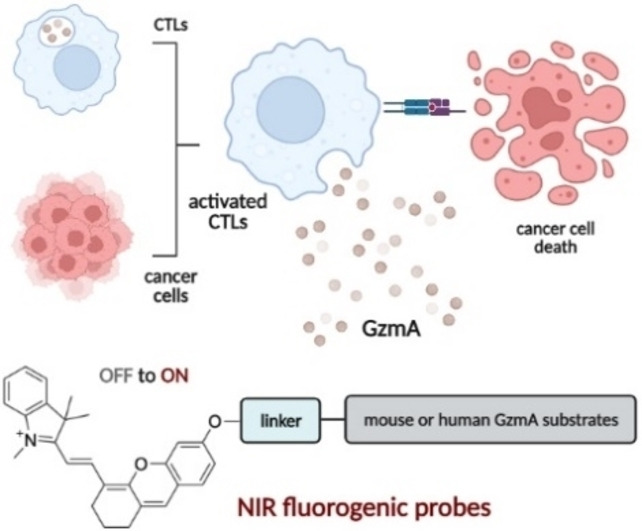

## Introduction

The immune system plays a central role in host defense against infections and tumors.^[1]^ Among its components, cytotoxic T lymphocytes (CTLs) and natural killer (NK) cells recognize and eliminate cancer cells, and their abundance in tumors is regarded as a positive prognostic indicator in cancer patients.^[2]^ The activity of CTLs and NK cells can be reinvigorated by immunotherapies, which hold promise as anticancer strategies; however, there is large variability in how cancer patients respond to immunotherapy, making the optimization of personalized treatments difficult.^[3]^ Imaging the recruitment of CTLs in tumors can inform partially about immune reinvigoration, yet most modalities (e.g., CT scans) cannot inform about the functional cross‐talk between immune and cancer cells.^[4]^ Activity‐based probes to monitor CTL and NK cell function would accelerate the design of anticancer therapies.

Different approaches can measure tumor cell proliferation and viability, such as ^51^Cr release, lactate dehydrogenase or tetrazolium dye‐based assays^[5]^ but these do not typically provide specific readouts of CTL or NK‐mediated cytotoxicity. CTLs and NK cells employ a myriad of strategies to destroy cancer cells,^[6]^ including the secretion and delivery of serine proteases (e.g., granzymes).^[7]^ Granzymes are stored in cytolytic granules,^[8]^ with granzyme A (GzmA) and granzyme B (GzmB) being the most abundant subtypes.^[7a]^ The expression levels of GzmA can be determined using antibodies,^[9]^ but such constructs do not typically differentiate between the active and inactive forms of the enzyme. Because granzymes can be stored as inactive enzymes before CTLs and NK cells recognize cancer cells, the functionality of GzmA and GzmB can correlate to cancer cell engagement and cytotoxic capacity.^[10]^ To this end, several optical probes have been described to detect active granzymes.^[11]^ Probes targeting GzmB include substrate and inhibitor‐based structures, whereas most probes for GzmA are fluorescently‐labeled phosphonate inhibitors that react with GzmA to form covalent fluorescent conjugates.^[12]^ Fluorescent inhibitors can have limited sensitivity, which hinders the optical detection of enzymes at physiological concentrations, whereas substrate‐based probes containing FRET pairs^[13]^ or fluorogenic reporters^[14]^ can amplify the fluorescence signals and enable real‐time imaging in multiple biological systems (e.g., cells, lysates or supernatants).

**Figure 1:**
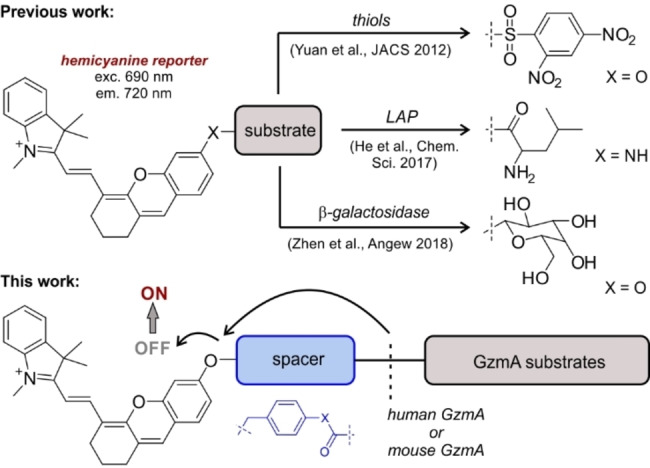
Representative hemicyanine probes for different biological targets[^15a^, ^16a^, ^16b^] and our adaptation to the rational design of GzmA substrate probes.

Among the different fluorogenic scaffolds that can be employed for the preparation of activity‐based reporters, hemicyanines display good biocompatibility, near‐infrared (NIR) excitation and emission wavelengths, and reasonable fluorescence quantum yields (Figure 1).^[15]^

Hemicyanine fluorophores have been described for targeting different biomolecules and enzymes,^[16]^ but there are no examples of NIR fluorescent substrates to image active GzmA resulting from the interactions between immune cells and cancer cells. Herein, we rationally designed fluorogenic probes to detect physiological levels of active GzmA in mouse and human cells and tissues, and to image CTL reinvigoration against cancer cells in real time.

## Results and Discussion

**Figure 2:**
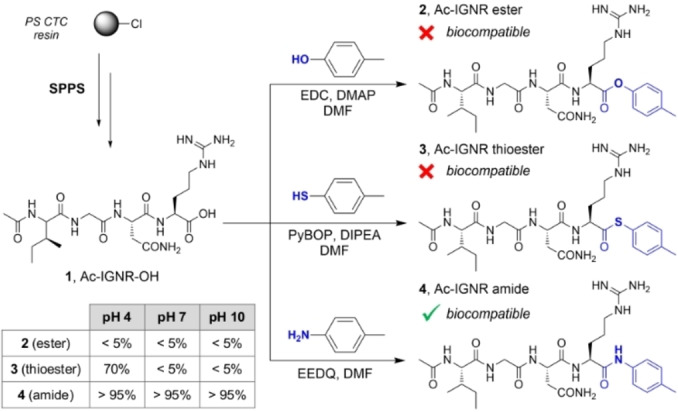
Solid‐phase peptide synthesis (SPPS) of the sequence Ac‐IGNR‐OH (compound **1**) and corresponding analogues containing spacers with different connectivities. The synthesis of compounds **1**–**4** is described in the Supporting Information. The table shows the stability of compounds **2**–**4** as determined by HPLC‐MS after incubation for 2 h at 37 °C in aqueous buffers of different pH values.

We started the synthesis of fluorogenic GzmA substrates by using the sequence Ac‐Ile‐Gly‐Asn‐Arg (Ac‐IGNR), which was identified by Craik et al. using positional scanning^[17]^ but has not been used to prepare NIR fluorogenic substrates. Due to the dimeric structure of human GzmA (hGzmA) and the close proximity of the dimer interface to the binding pockets, the accessibility to the active site of hGzmA is limited.^[17]^ To overcome this potential steric hindrance, we included a spacer between the peptide substrate and the hemicyanine reporter to: 1) facilitate the reaction between the IGNR sequence and the enzyme, and 2) quench the emission of the hemicyanine core so that strong fluorescent signals were only detected after enzymatic cleavage (Figure 1).^[18]^ To optimize the structure of the spacer, we compared the biocompatibility of three Ac‐IGNR‐based derivatives including different connecting heteroatoms (i.e., N, O and S) that formed amide, ester and thioester bonds, respectively (Figure 2). The only precedent of these spacers in GzmA substrates relates to the thioester Z‐Arg‐SBzl, a commercially available reagent to measure generic tryptase‐like activity by absorbance measurements.^[19]^

First, we synthesized a large batch (>400 mg) of Ac‐IGNR‐OH using standard protocols for the solid‐phase synthesis of fluorescent peptides^[20]^ in polystyrene resin derivatized with a 2‐chlorotritylchloride linker (PS‐CTC). Next, we coupled the tetrapeptide Ac‐IGNR‐OH (compound **1**, Figure 2) to different aryl groups to generate the isosteric ester, thioester and amide analogues (compounds **2**, **3** and **4** respectively, Figure 2). After isolation of all compounds by semi‐preparative HPLC with purities >95 %, we analyzed their chemical stability in aqueous media in the absence of hGzmA. As shown in Figure 2, we found that compounds **2** and **3** were completely hydrolyzed in Tris buffer (pH 7.4) whereas compound **4** was fully stable under the same experimental conditions (purity >95 %). We also tested their chemical integrity at different pH values because GzmA can be stored in slightly acidic cytotoxic granules inside CTLs.^[21]^ In these experiments, we observed that the amide compound **4** was stable in basic and acid media, whereas the thioester **3** was more stable than the carboxylic ester analogue **2** at acidic pH (Figure 2). Finally, we confirmed by HPLC‐MS that the Ac‐IGNR amide derivative could be cleaved by recombinant hGzmA (Figure S1), thus we focused on this scaffold for the generation of fluorogenic GzmA substrates.

**Figure 3:**
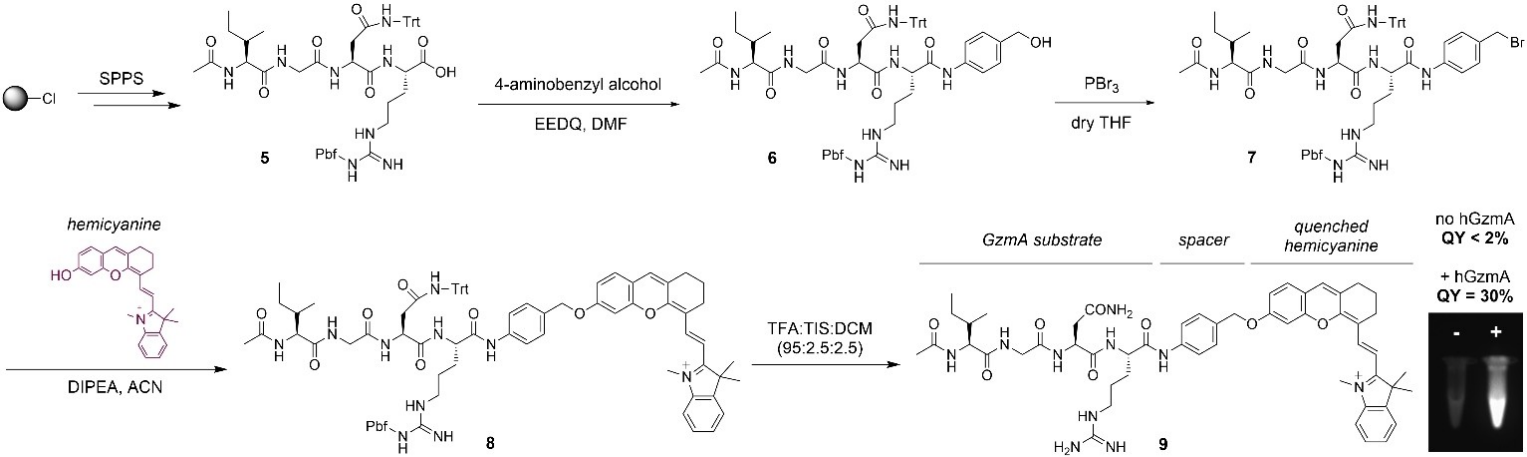
Synthetic scheme for the preparation of fluorogenic substrates for hGzmA. Relative fluorescence quantum yields (standard: IR‐125) of the compound **9** before and after incubation with hGzmA (20 nM) in Tris buffer (pH 8). Pictograms of solutions of the compound **9** (50 μM) in the absence (left) or presence (right) of hGzmA under 650 nm excitation.

After the selection of a suitable spacer, we synthesized the protected sequence Ac‐IGN(Trt)R(Pbf)‐OH, where the side chains of Asn and Arg residues were protected with trityl (Trt) and 2,2,4,6,7‐pentamethyldihydrobenzofuran‐5‐sulfonyl (Pbf) groups respectively, to enable site‐specific conjugation of the hemicyanine fluorophore at the C‐terminal end of the peptide (Figure 3). The protected peptide **5** was isolated as a carboxylic acid and then coupled to 4‐aminobenzyl alcohol spacer using N‐ethoxycarbonyl‐2‐ethoxy‐1,2‐dihydroquinoline (EEDQ) to yield the peptide‐spacer **6** (Figure 3). Next, we replaced the hydroxyl group of compound **6** with a better leaving group (i.e., bromide) by treatment with PBr_3_ (compound **7**) and performed the nucleophilic substitution with the hemicyanine fluorophore to yield compound **8** (synthesis of the hemicyanine described in the Scheme S1 in the Electronic Supporting Information). Finally, we removed the Trt and Pbf protecting groups by treatment with trifluoroacetic acid (TFA) and isolated the final compound **9** in high purity (>95 %) after purification by semi‐preparative HPLC. The synthetic protocols for all intermediates together with their chemical characterization are included in the Electronic Supporting Information.

**Figure 4:**
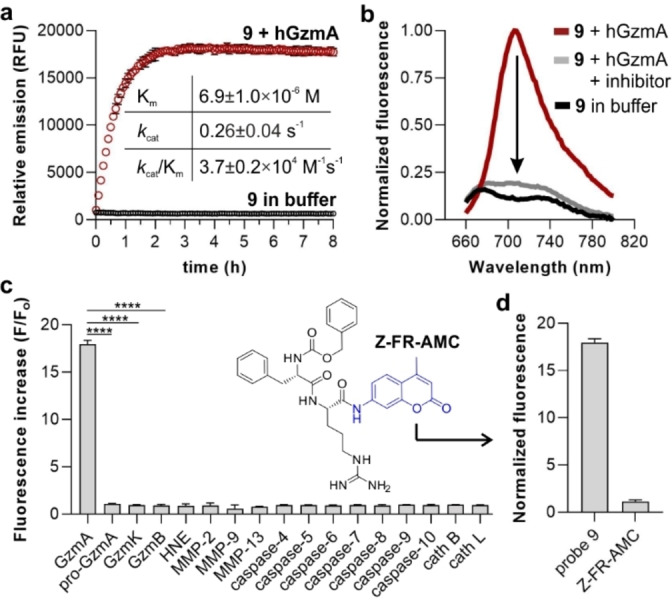
In vitro characterization of compound **9** in response to hGzmA. a) Time‐lapse fluorescence emission (725 nm) of compound **9** (20 μM) in the absence (black circles) or presence (red circles) of hGzmA (20 nM) in Tris buffer (pH 8) at 37 °C. Data as means±SD (*n*=3). Inset) Kinetic constants of compound **9** after reaction with hGzmA calculated using the Michaelis–Menten equation. Data presented as means±SD (*n*=3). b) Fluorescence spectra of compound **9** (20 μM) after incubation with hGzmA (20 nM, red) and in the absence (black) or presence of 3,4‐dichloroisocoumarin (100 μM, grey) in Tris buffer (pH 8). Representative plots from 3 independent experiments. c) Fluorescence fold increase of compound **9** (20 μM) after incubation with different proteases (all at 20 nM) at 37 °C for 60 min. Data presented as means±SD (*n*=3) and p values obtained from one‐way ANOVA (**** for p<0.0001). Inset) Chemical structure of the commercial Z‐FR‐AMC. d) Fluorescence enhancement of compound **9** and Z‐FR‐AMC (both at 20 μM) after incubation with hGzmA (20 nM) in Tris buffer (pH 8) at 37 °C for 60 min. Data presented as means±SD (*n*=3).

Next, we examined the capabilities of compound **9** as a fluorogenic probe to detect active hGzmA. We monitored the time‐lapse fluorescence emission of compound **9** in the absence or presence of active recombinant hGzmA and observed bright NIR emission >700 nm after the enzymatic reaction, with around 20‐fold fluorescence increase (Figure 4). Notably, compound **9** showed response within minutes and a stable fluorescence readout for several hours, making it compatible with multiple imaging assays (Figure 4). We also analysed the fluorescence emission of compound **9** in the presence of hGzmA and the serine protease inhibitor 3,4‐dichloroisocoumarin,^[22]^ which showed >90 % decrease of the fluorescence signal, confirming the reactivity of compound **9** with the active enzyme. Finally, we performed HPLC‐MS analysis of the reaction products derived from the incubation of compound **9** with hGzmA and corroborated that the enzymatic cleavage happens after the Arg residue with concomitant release of the 4‐aminobenzyl alcohol spacer and the hemicyanine fluorophore (Figure S2).

We also evaluated the selectivity of compound **9** by screening several proteases found in CTLs, NK cells and other tumor‐related immune cells, namely inactive hGzmA (pro‐hGzmA), other granzymes expressed by CTLs and NK cells (GzmB and GzmK), caspases as well as enzymes secreted by immune cells commonly found in tumors (e.g., human neutrophil elastase (HNE) from neutrophils, matrix metalloproteinases (MMPs) and cathepsins (cath B and cath L) from macrophages (Figure 4). In these experiments, we observed that compound **9** was reacting preferentially with active hGzmA compared to other proteases commonly found in the tumor microenvironment. Lastly, we evaluated the sensitivity of compound **9** to measure physiological levels of GzmA, which are typically found within the low nanomolar range in biological samples.^[23]^ The limit of detection of compound **9** for hGzmA was determined to be around 400 pM (Figure S3), confirming its suitability to detect active GzmA under physiological conditions.

Next, we determined the kinetic properties of compound **9** as a substrate for hGzmA using Michaelis–Menten's equation (Figures 4 and S4). In these experiments, we measured the maximum rate (*v*_max_), as well as the Michaelis (*K*_m_) and the catalytic rate (*k*_cat_) constants. Notably, compound **9** displayed a high catalytic efficiency for hGzmA with *k*_cat_/*K*_m_ ratios around 37 000 M^−1^ s^−1^. To the best of our knowledge, these are the highest *k*_cat_/*K*_m_ values reported to date for NIR GzmA substrates and outperform previously reported probes.^[17]^ This improvement in reactivity can be partially attributed to the enhanced accessibility of the scissile bond in compound **9** due to the addition of the 4‐aminobenzyl alcohol spacer. We confirmed this hypothesis by preparing aminomethyl coumarin (AMC) substrates containing the same Ac‐IGNR sequence but without the spacer (Figure S5), which showed significantly reduced reactivity (Figures S6 and S7). Finally, we also compared the performance of compound **9** to the commercial substrate Z‐FR‐AMC (Figure 4). In addition to exhibiting longer emission wavelengths (e.g., 445 nm for Z‐FR‐AMC, ≈720 nm for compound **9**), compound **9** displayed 18‐fold higher fluorescence increase than Z‐FR‐AMC under the same experimental conditions (Figure 4).

**Figure 5:**
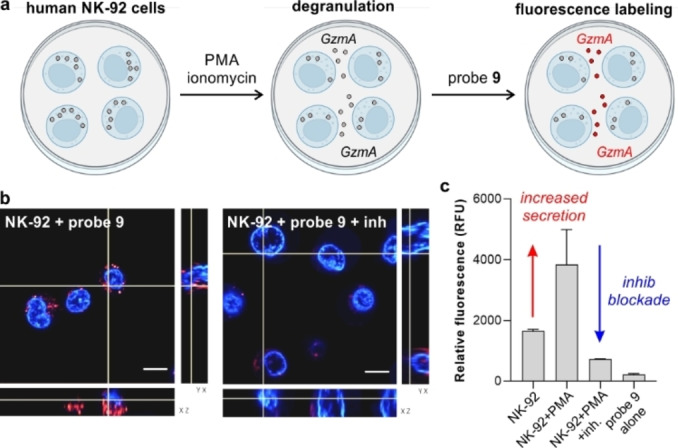
Detection of active GzmA in human NK‐92 cells. a) Schematic illustration showing that activation of NK‐92 cells induces degranulation and secretion of active GzmA to the extracellular space for subsequent labeling with compound **9**. b) Representative fluorescence confocal microscopy Z‐stack images from 3 independent experiments of live NK‐92 cells upon incubation with compound **9** (red, 5 μM) for 1 h and Hoechst (blue) for nuclear counterstaining. 3,4‐dichloroisocoumarin (5 μM) was used as a GzmA inhibitor for 2 h prior to labeling. c) Relative fluorescence emission (725 nm) of cell supernatants collected from stimulated and non‐stimulated cells. Data presented as means±SD (*n*=3).

Encouraged by the high reactivity of compound **9**, we evaluated its potential application to detect active GzmA secreted by immune cells. NK cell‐based therapies are regarded as potential anticancer treatments due to their ability to secrete granzymes to the extracellular space and cause cytotoxicity in cancer cells.^[24]^ The levels of secreted GzmA by NK cells are typically measured by ELISA, although these assays do not distinguish between active and inactive enzymes. To test the utility of compound **9** to measure active GzmA in cell supernatants, we performed degranulation assays in human NK‐92 cells (e.g., a patient‐derived cell line that expresses higher levels of GzmA than healthy NK cells)^[25]^ and also tested GzmA‐negative cells (e.g., HaCaT keratinocytes, Figure S8). NK‐92 cells were stimulated with phorbol myristate (PMA) and ionomycin (Figure 5).^[26]^ Cell supernatants were collected and incubated with compound **9** for 1 h, followed by spectroscopic measurements. As shown in Figure 5, we observed significantly brighter fluorescence in supernatants from stimulated NK‐92 cells when compared to non‐stimulated cells, and we confirmed that such emission was blocked by the protease inhibitor 3,4‐dichloroisocoumarin. Previous reports indicate that granzyme activity can be detected inside NK‐92 cells^[11b]^ and that granzymes can be found within activated immune cells due to intracellular granular leakage,^[27]^ even though granzyme activity is known to be reduced in acidic granules. To study this observation, we performed Z‐stack live‐cell confocal microscopy in NK‐92 cells after incubation with compound **9**. As shown in Figure 5 and Figure S9, compound **9** displayed bright intracellular fluorescence in NK‐92 cells, with signals being reduced in the presence of 3,4‐dichloroisocoumarin. Whereas further studies in non‐transformed NK cells might be necessary to analyze the behavior of compound **9** in non‐transformed healthy effector cells, these results suggest that compound **9** can also be employed for fluorescence microscopy of human NK‐92 cells.

**Figure 6:**
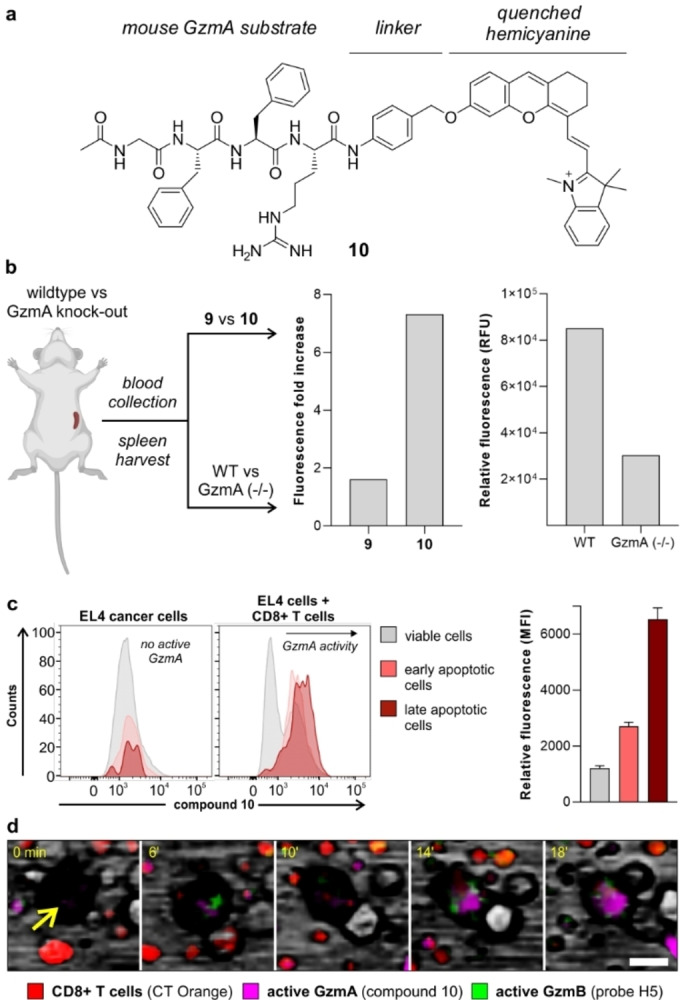
Compound **10** is a fluorogenic substrate for imaging active mGzmA in ex vivo mouse tissues and live CD8+ T cell‐cancer cell co‐cultures. a) Chemical structure of compound **10**. b) Fluorescence emission signals (λ_exc_: 680 nm) from two independent experiments including sera from septic mice incubated with compounds **9** and **10** (both at 20 nM, left panel) and spleen lysates from infected wild‐type (WT) mice and infected GzmA knock‐out mice after incubation with compound **10** (20 nM, right panel). c) Representative histograms from flow cytometric analysis of EL4 cancer cells before and after co‐culture with CD8+ T cells and incubation with compound **10** (2.5 μM). Mean fluorescence intensities are presented as means±SD (*n*=3). d) Representative snapshots of time‐lapse fluorescence microscopy of live co‐cultures of CD8+ T cells (red, counterstained with CellTracker^TM^ Orange) and EL4 cancer cells (yellow arrow) where GzmA (compound **10**, magenta, 3 μM) and GzmB (probe H5, green, 2.5 μM) activities were monitored upon antigen‐driven interaction (Movie S1 in the Electronic Supporting Information). Scale bar: 10 μm.

To further evaluate the potential of fluorogenic hemicyanine probes to detect active GzmA, we tested them in wild‐type C57BL/6 mice expressing GzmA and knock‐out GzmA (−/−) mice where the enzyme is not present. Previous studies have reported that mouse GzmA (mGzmA) and hGzmA do not share the same substrate preferences,^[17b]^ therefore we synthesized the new compound **10** (Figure 6) as a mouse‐reactive analogue including the same spacer and hemicyanine fluorophore but the mGzmA‐reactive peptide sequence Ac‐Gly‐Phe‐Phe‐Arg (Ac‐GFFR).^[17b]^ Similar to compound **9** (Figure 3), the preparation of compound **10** was performed by solid‐phase synthesis of the tetrapeptide Ac‐GFFR(Pbf)‐OH and subsequent derivatization with 4‐aminobenzyl alcohol and the hemicyanine fluorophore to isolate the final compound in high purity (>95 %) after HPLC purification (for synthetic and characterization details, see Scheme S2 in the Electronic Supporting Information). We performed HPLC‐MS analysis of the reaction between compound **10** and mGzmA to corroborate that the enzymatic cleavage of the substrate happens after the Arg residue (Figure S10).

We compared the reactivity of compounds **9** and **10** using a model of sepsis, where the levels of GzmA in blood are elevated.^[28]^ Briefly, we incubated sera from septic mice with compounds **9** or **10**, observing much brighter signals for the latter, with over 7‐fold fluorescence increase (Figure 6). This result corroborates that the peptide sequence GFFR is more reactive for mGzmA than the IGNR sequence. We also confirmed that compound **10** did not cross‐react with other proteases (e.g., cathepsins, Figure S11). Next, we stimulated the in vivo expression of granzymes in both wild‐type (WT) and GzmA knock‐out mice by administration of lymphocytic choriomeningitis virus (LCMV), a widely used model to study adaptive immunity.^[29]^ Briefly, we harvested spleens and livers from both infected WT and infected GzmA knock‐out mice, and incubated their lysates with compound **10** under the same experimental conditions. As shown in Figure 6 and Figure S12, we observed brighter fluorescence signals in WT lysates than in GzmA knock‐out lysates, indicating the ability of compound **10** to preferentially react with active mGzmA.

In view of these results, we decided to employ compound **10** as a fluorogenic probe for real‐time measurements of GzmA activity in cancer cells that are targeted by antigen‐specifically activated CTLs. For these experiments, we utilized CD8+ T cells containing OT‐I transgenic receptors that target the SIINFEKL antigen and co‐cultured them with SIINFEKL‐treated mouse EL4 cancer cells. First, we corroborated that compound **10** was not activated inside other immune cells with high proteolytic activity^[30]^ (e.g., mouse RAW264.7 macrophages, Figure S13) and used flow cytometry to compare the fluorescence staining of cancer cells before and after they had been co‐cultured with CD8+ T cells. In these experiments, we did not observe major differences in the fluorescence signals of live and apoptotic mono‐cultured cancer cells; however, cancer cells that had been co‐cultured and attacked by CTLs showed clear differences in fluorescence labeling by the compound **10**, with higher levels of active GzmA being found in apoptotic ‐both early and late apoptosis‐ than live cancer cells (Figure 6). This observation highlights the presence of active GzmA in cancer cells upon antigen‐driven recognition by adaptive immune cells.

Finally, we also used this co‐culture system to visualize the longitudinal activity of both GzmA and GzmB in real time (Figure 6). Our group has recently reported the probe H5 as a green fluorescent reporter for active GzmB,^[11d]^ with non‐overlapping emission with compound **10** (Figure S14). CD8+ T cells were first labeled with CellTracker^TM^ Orange as a counterstain, and then co‐cultured with cancer cells in the presence of compound **10** and probe H5 to monitor the activation and localization of the two granzymes by time‐lapse fluorescence microscopy. Real‐time images showed almost simultaneous bursts of activity for both enzymes in EL4 cancer cells (Figure 6 and Movie S1), suggesting that CTLs can release both active GzmA and GzmB upon antigen‐driven recognition. To the best of our knowledge, this study represents one of the first examples of real‐time multiplexed imaging of granzyme activities in co‐cultures of cancer cells and adaptive immune cells.

## Conclusion

We have rationally designed NIR fluorogenic substrate‐based probes for imaging human GzmA (compound **9**) and mouse GzmA (compound **10**). These probes consist of species‐selective tetrapeptide sequences that are connected to a turn‐on hemicyanine fluorophore via a biocompatible self‐immolative linker. We demonstrated that these activatable probes produce bright NIR fluorescence after cleavage by GzmA but not in the presence of GzmB, GzmK or other proteases. Compound **9** displays high sensitivity and catalytic efficiency for hGzmA, outperforming commercial reagents and previously reported fluorophores. Compound **10** exhibits bright labeling of spleen lysates from stimulated WT mice but not from stimulated GzmA knock‐out mice. We have used the probes for real‐time imaging of active GzmA secreted by reinvigorated human NK‐92 cells (compound **9**) and antigen‐driven recognition of mouse CTLs in co‐cultures with cancer cells (compound **10**). These probes will enable future imaging studies of adaptive immunity and accelerate the optimization of CTL and NK‐based anticancer immunotherapies.

## Conflict of interest

The authors declare no conflict of interest.

1

## Supporting information

As a service to our authors and readers, this journal provides supporting information supplied by the authors. Such materials are peer reviewed and may be re‐organized for online delivery, but are not copy‐edited or typeset. Technical support issues arising from supporting information (other than missing files) should be addressed to the authors.

Supporting Information

Supporting Information

## Data Availability

The data that support the findings of this study are available from the corresponding author upon reasonable request.
