# Supplementary material for: Fluorogenic Granzyme A Substrates Enable Real‐Time Imaging of Adaptive Immune Cell Activity
Source: Angew Chem Weinheim Bergstr Ger. 2023 Jan 16;135(8):e202216142. doi: 10.1002/ange.202216142 (PMC10953043; doi:10.1002/ange.202216142)
Supplement: Supplementary file 1 — Supporting Information [file ANGE-135-0-s001.pdf]

Angewandte Chemie
Eine Zeitschrift der Gesellschaft Deutscher Chemiker

Supporting Information

Fluorogenic Granzyme A Substrates Enable Real-Time Imaging of Adaptive Immune Cell Activity

*Z. Cheng, E. J Thompson, L. Mendive-Tapia, J. I Scott, S. Benson, T. Kitamura, A. Senan-Salinas, Y. Samarakoon, E. W Roberts, M. A Arias, J. Pardo, E. M Galvez, M. Vendrell**

Electronic Supporting Information

Table of Contents

Supplementary Figures

Experimental Details

NMR Spectra

Supplementary References

Supplementary Figures

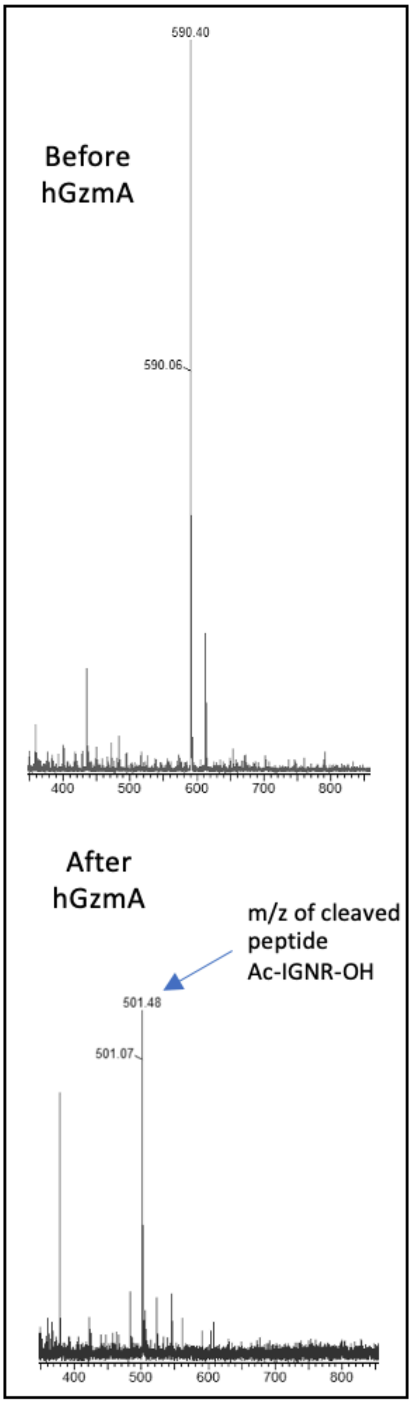

Figure S1. Mass spectra of compound 4 before (top) and after (below) reaction with hGzmA. Compound **4** (10 μM) was incubated with hGzmA (20 μM) in Tris buffer (pH 8) at 37°C for 2 h. m/z (calc. for compound **4**): 589.3, m/z (calc. for cleaved peptide): 500.3.

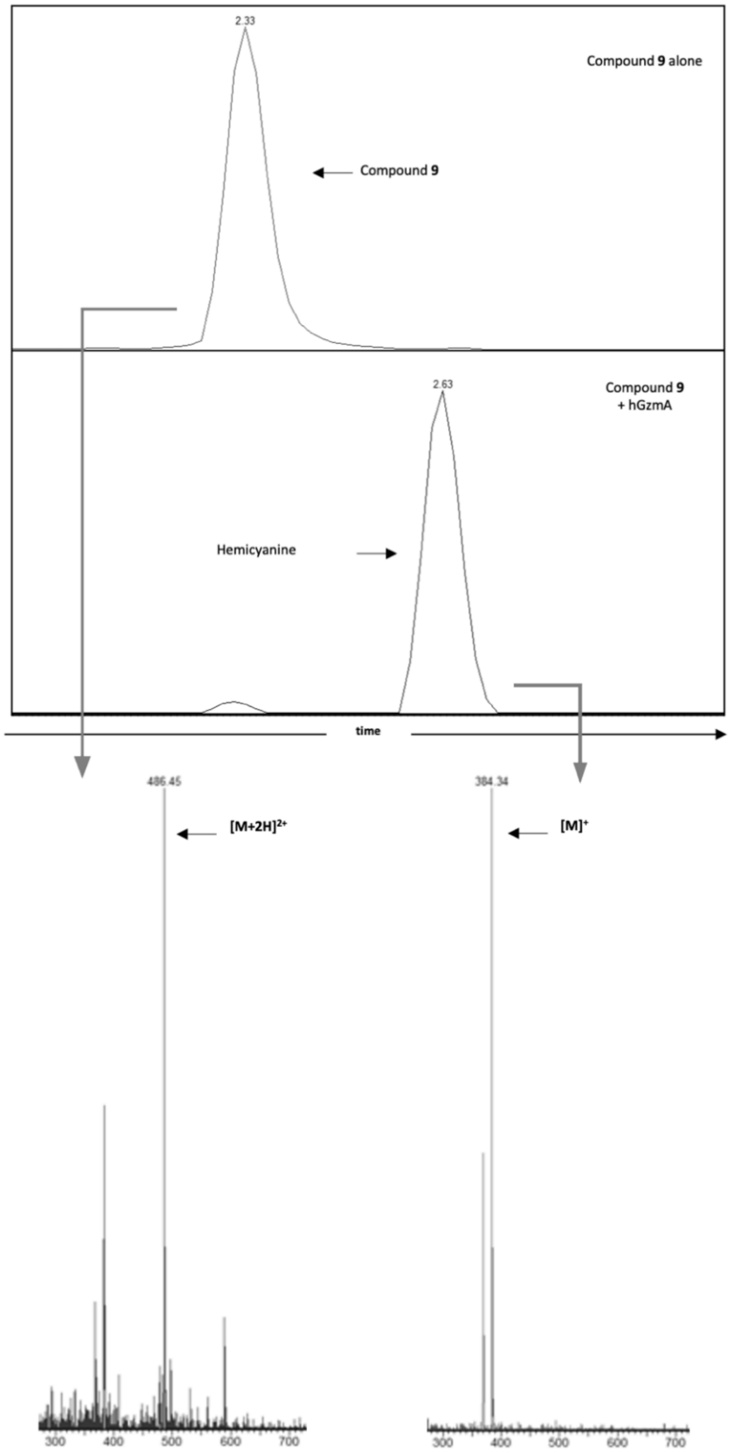

Figure S2. HPLC-MS traces of compound 9 before and after reaction with hGzmA. Compound **9** (20 μM) was incubated with hGzmA (20 nM) in Tris buffer (pH 8) at 37°C for 90 min. HPLC traces were monitored at 650 nm before and after the reaction. m/z (calc. for compound **9**): 971.5, m/z (calc. for hemicyanine): 384.2.

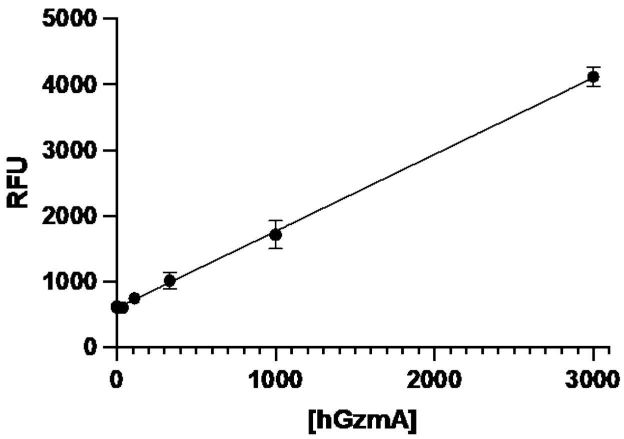

Figure S3. Limit of detection of compound 9 for hGzmA. The hGzmA concentrations used were 3000, 1000, 333, 111, 37, 12, 4 and 1.4 pM. Enzymatic assays were performed with compound **9** (20 μM) in Tris buffer (pH 8) at 37 °C. Data presented as means±SD (n=3). LoD = 472 pM.

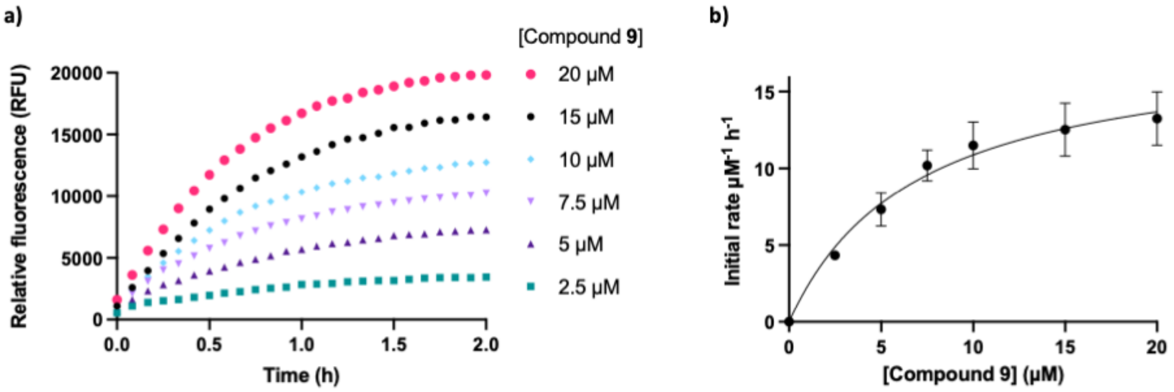

Figure S4. Kinetics of the compound 9 upon reaction with hGzmA. a) Representative (from 3 independent experiments) time-lapse fluorescence emission derived from the reaction of hGzmA (20 nM) and compound **9** at different concentrations [2.5 μM, 5 μM, 7.5 μM, 10 μM, 15 μM, 20 μM]. b) Initial rates (V_0) were plotted against different concentrations of compound **9** (2.5, 5, 7.5, 10, 15 and 20 μM) when incubated with hGzmA (20 nM) in Tris buffer (pH 8) at 37°C. Data presented as means±SD (n=3).

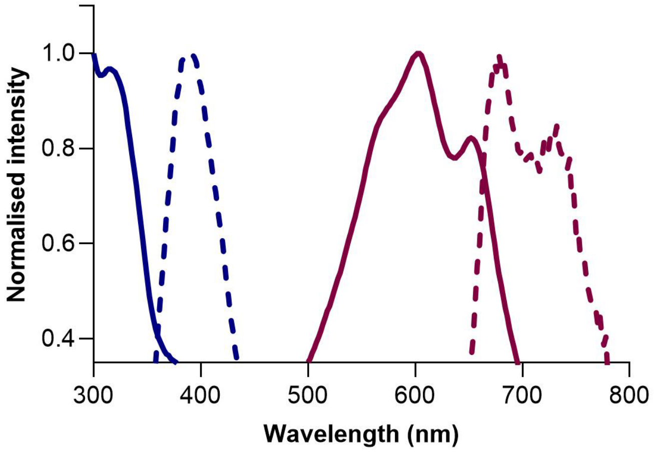

Figure S5. Photophysical properties of Ac-IGNR-AMC and compound 9. Absorbance (solid) and emission (dashed) spectra of **Ac-IGNR-AMC** (20 μM, blue) and compound **9** (20 μM, red) when dissolved in Tris buffer (pH 8).

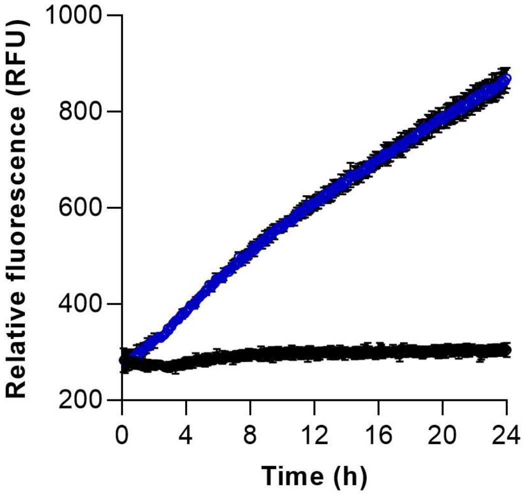

Figure S6. Reactivity analysis for the spacer-free probe Ac-IGNR-AMC. Time-lapse fluorescence emission (460 nm, exc: 380 nm) of **Ac-IGNR-AMC** (20 μM) in the absence (black) or presence (blue) of hGzmA (20 nM) in Tris buffer (pH 8) at 37 °C. Data presented as means±SD (n=3).

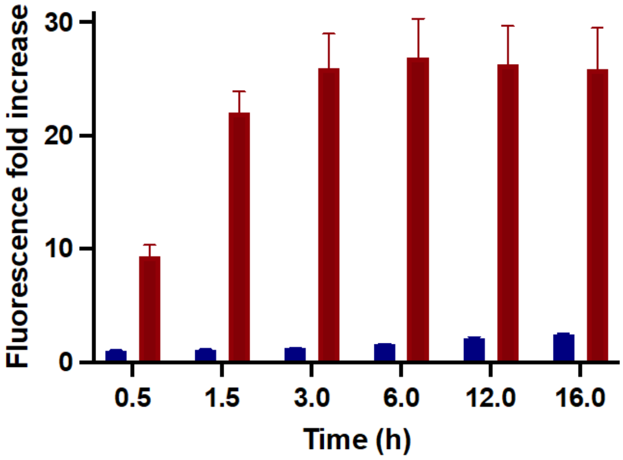

Figure S7. Comparative reactivity against hGzmA for Ac-IGNR-AMC and compound 9. Fluorescence fold increase of **Ac-IGNR-AMC** (20 μM, blue) and compound **9** (20 μM, red) over time when incubated with hGzmA (20 nM) in Tris buffer (pH 8). Data presented as means±SD (n=3).

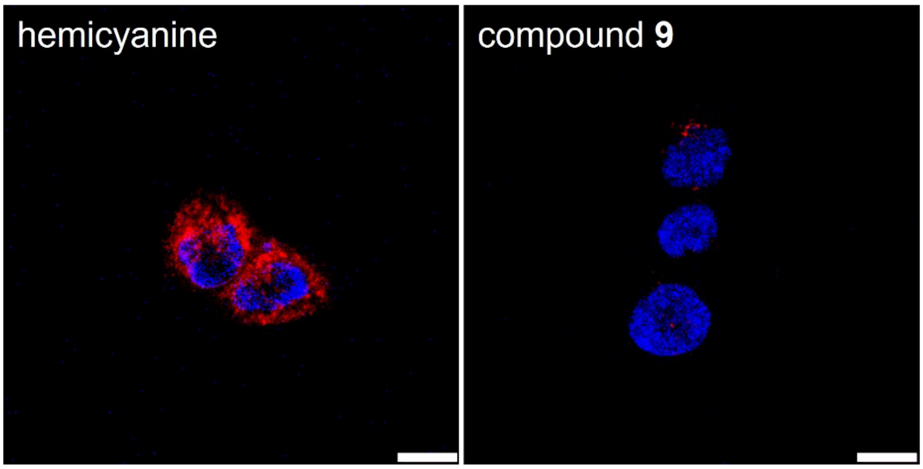

Figure S8. Fluorescence confocal microscopy images of live HaCaT cells upon incubation with compound 9. Live HaCaT cells were incubated with compound **9** (red, 5 μM) and hemicyanine (red, 5 μM) for 1 h. Hoechst (blue) was used for nuclear counterstaining. Scale bar: 10 μm.

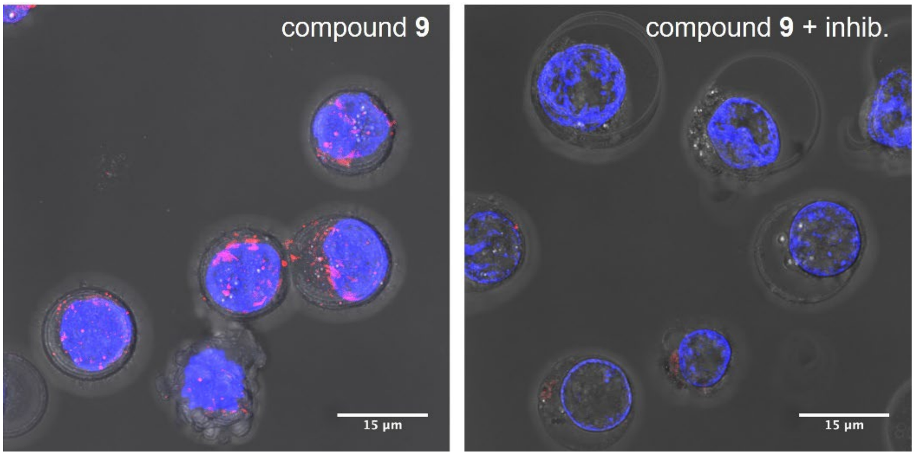

Figure S9. **Brightfield and fluorescence confocal microscopy merged images of live NK-92 cells upon incubation with compound 9.** Representative microscopy images from 3 independent experiments of live NK-92 cells upon incubation with compound **9** (red, 5 µM) for 1 h and Hoechst (blue) for nuclear counterstaining. 3,4-dichloroisocoumarin (5 µM) was used as a GzmA inhibitor for 2 h prior to labeling. Scale bar: 15 µm.

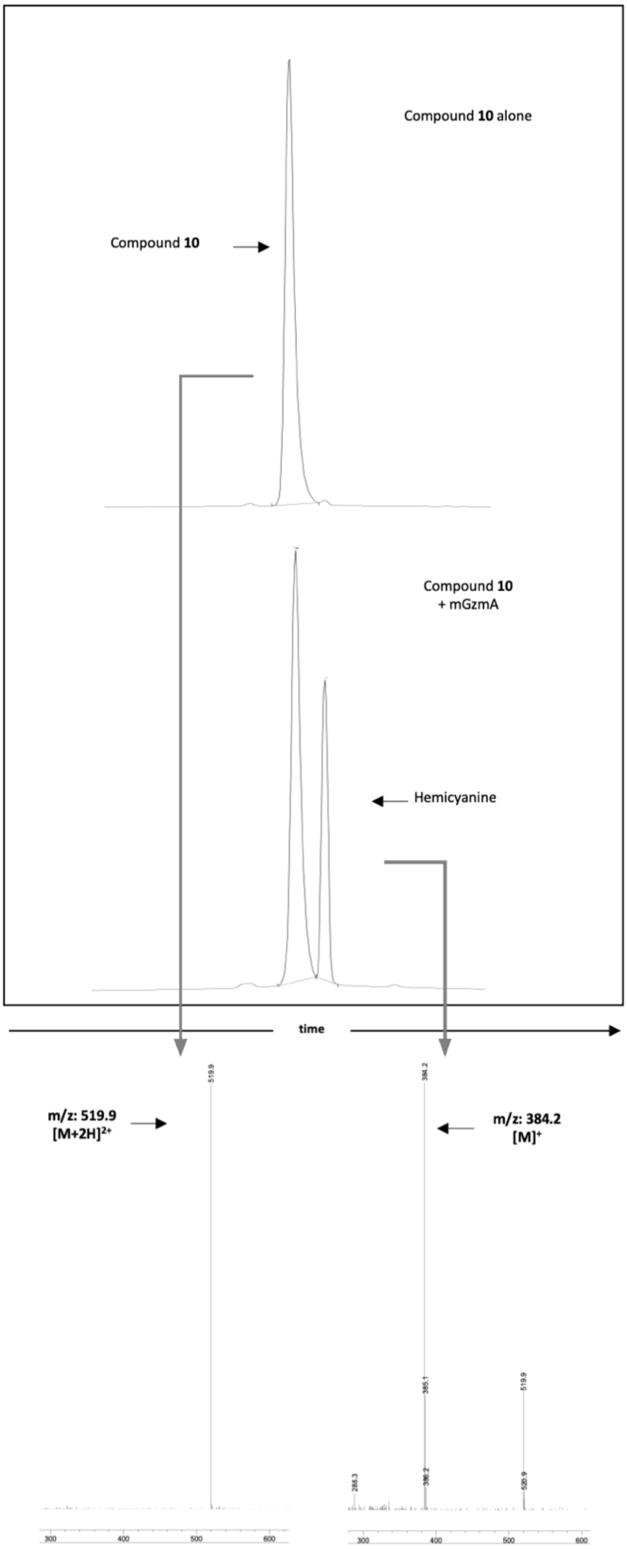

Figure S10. HPLC-MS traces of compound 10 before and after reaction with mGzmA. Compound **10** (20 µM) was incubated with mGzmA in Tris buffer (pH 8) at 37°C for 24 h. HPLC traces were monitored at 650 nm before and after the reaction. m/z (calc. for compound **10**): 1038.5, m/z (calc. for hemicyanine): 384.2.

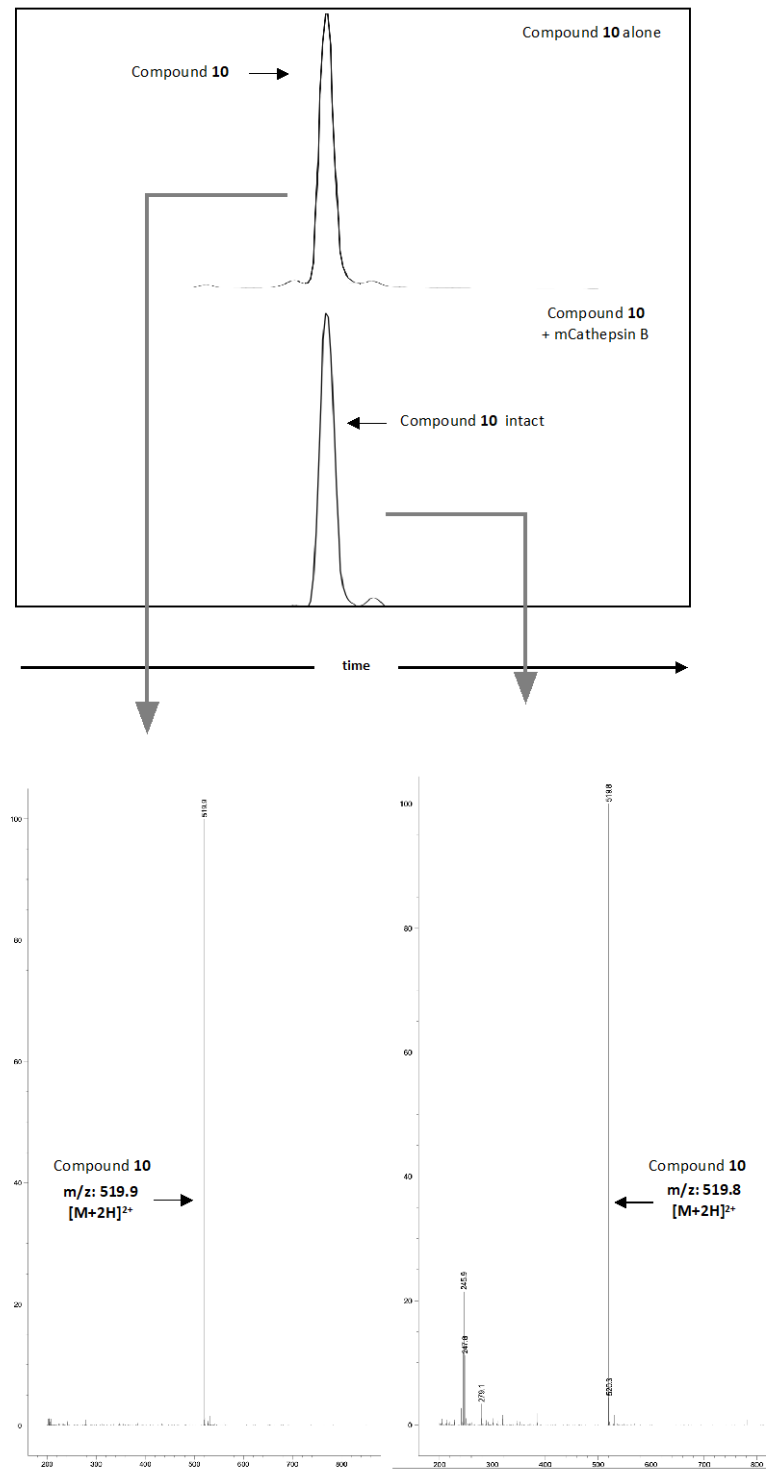

Figure S11. HPLC-MS traces of compound 10 before and after reaction with mouse cathepsin B. Compound **10** (20 μM) was incubated with mouse cathepsin B in MES buffer (pH 5) at 37 °C for 24 h. HPLC traces were monitored at 650 nm before and after the reaction. m/z (calc. for compound **10**): 1038.5, m/z (calc. for hemicyanine): 384.2.

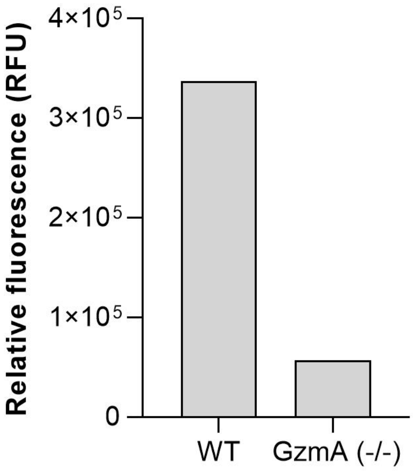

Figure S12. **Fluorescence analysis of compound 10 with liver lysates from infected wild-type mice and GzmA knock-out mice.** Fluorescence emission signals (680-750 nm) from two independent experiments including liver lysates from infected wild-type mice and infected GzmA knock-out mice after incubation with compound **10** (20 nM).

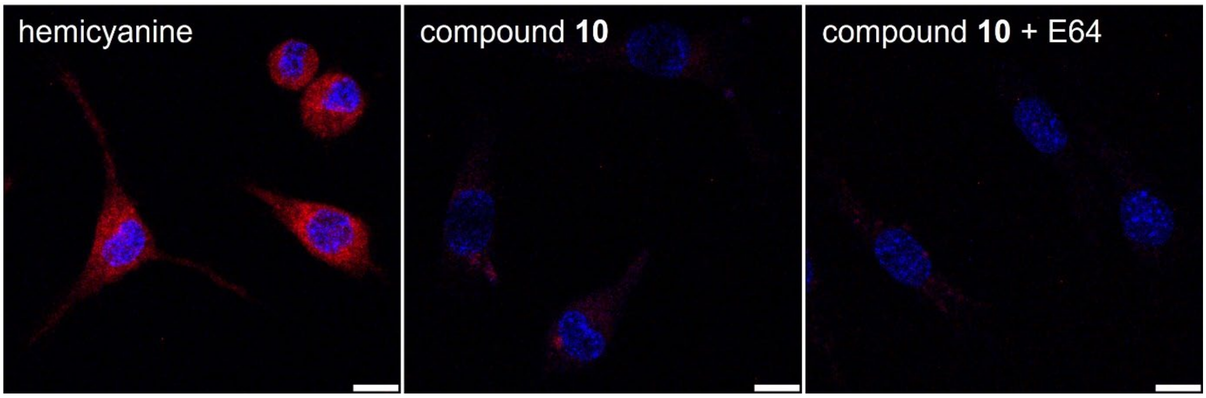

Figure S13. **Fluorescence confocal microscopy images of live RAW 264.7 cells upon incubation with compound 10.** Live RAW 264.7 cells were incubated with compound **10** (red, 5 µM) for 1 h and Hoechst (blue) was used for nuclear counterstaining. E-64 (1 µM) was used as a cathepsin inhibitor for 1 h prior to labeling. Scale bar: 10 µm.

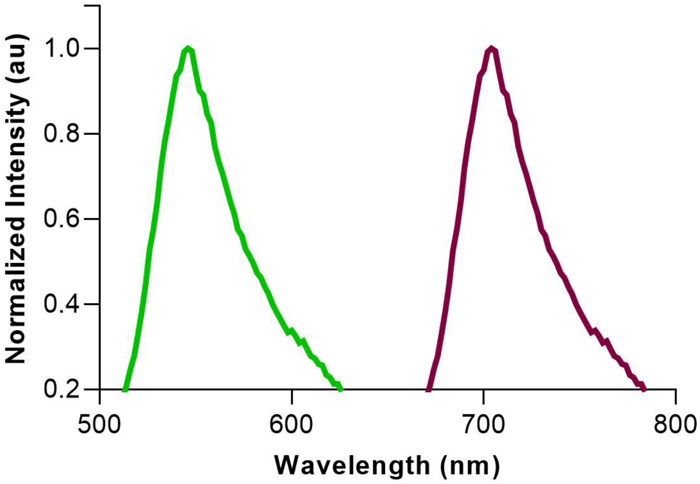

Figure S14. Fluorescence emission spectra of the fluorescent reporters of GzmA-reactive compound **10** (hemicyanine fluorophore, magenta) and GzmB-reactive probe **H5** (BODIPY-FL fluorophore, green).[1]

Movie Legends

Movie S1. Time-lapse fluorescence microscopy of live co-cultures of CD8+ T cells (counterstained with CellTracker^{TM} Orange, red) and EL4 cancer cells. Cells were incubated with compound **10** (3 μM, magenta for GzmA activity) and probe H5 (2.5 μM, green for GzmB activity). Movie recorded at 1 frame every 2 minutes.

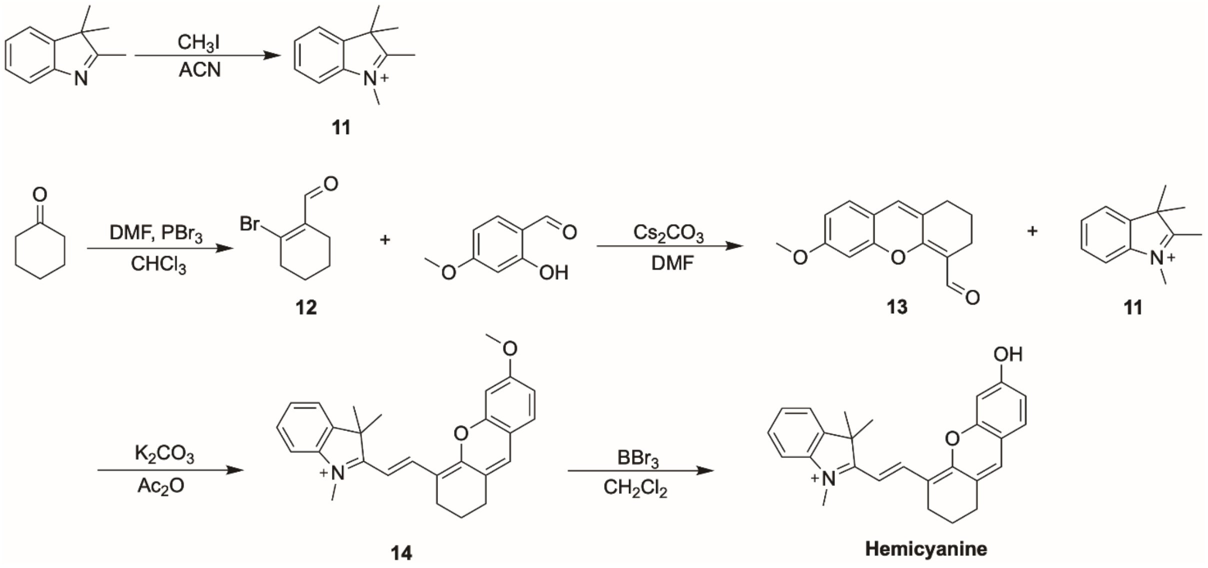

Scheme S1. Synthetic scheme for the preparation of the hemicyanine fluorophore. The hemicyanine fluorophore was adapted from reported procedures.[2]

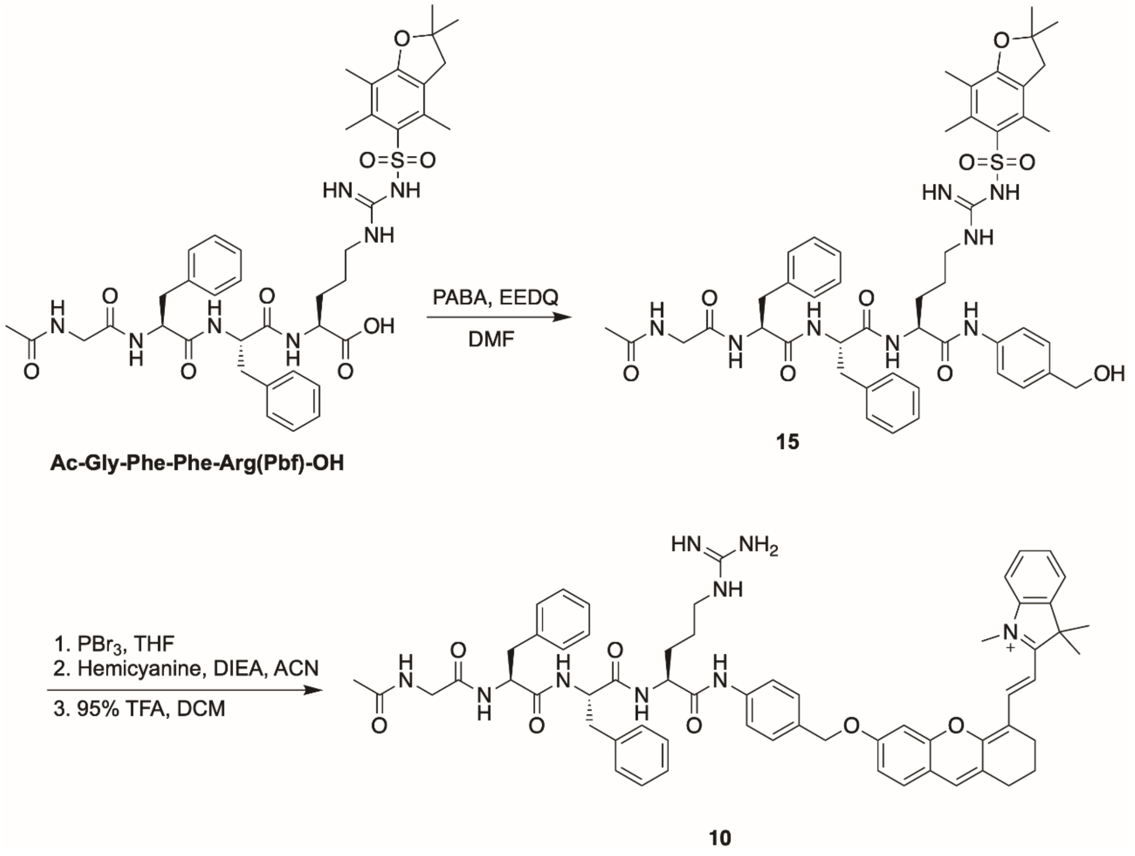

Scheme S2. Synthetic scheme for the preparation of compound **10**.

Experimental Details

Materials and methods. Chemicals and solvents were either AR grade or purified by standard techniques. Column chromatography (FC): silica gel Merck 60 (particle size 0.040-0.063 mm) or via Teledyne Isco, eluent systems as indicated. Fmoc-AA-OH were obtained from Iris Biotech GmbH (Fmoc-Ile-OH, Fmoc-Gly-OH, Fmoc-Asn(Trt)-OH, Fmoc-Arg(Pbf)-OH, Fmoc-Phe-OH). HATU, 2-ethoxy-1-ethoxycarbonyl-1,2-dihydroquinoline, 4-aminobenzyl-alcohol, boron tribromide, 4-methylbenzenethiol, 4-N,N'-(dimethylamino)pyridine were purchased from Sigma Aldrich. PyBOP and 7-amino-4-methylcoumarin were purchased from Alfa Aesar. N,N'-ethyldiisopropylamine was purchased from VWR International. p-Cresol was purchased from Fluorochem UK. EDC HCl was purchased from Novabiochem. Polystyrene resin with a 2-chlorotrityl chloride linker was obtained from Iris Biotech GmbH. MMP-2 and MMP-9 were purchased from Cambridge Biosciences. Recombinant human cathepsin B was purchased from Abcam. Recombinant hGzmA (pro-form) and recombinant mouse cathepsin B were purchased from Bio-Techne Ltd. Recombinant hGzmK was purchased from Generon Ltd. MMP-13, neutrophil elastase, caspase-5 and 10 were purchased from Merck Life Sciences. Recombinant hGzmA, human recombinant GzmB, human cathepsin L, caspase-4, 6, 7, 8 and 9 were purchased from Enzo Life Sciences and human IL-2 was purchased from Peprotech. Spectroscopic data was measured on a Synergy HT spectrophotometer (Biotek) and the data analysis was performed using GraphPad Prism 5.0. Peptide synthesis reactions and products were monitored by HPLC-MS using a HPLC Waters Alliance 2695 with a Kinetex C_{18} 50 × 4.6 mm column and a diode array detector. Eluents: H_2O (0.1% HCOOH) and ACN (0.1% HCOOH). Flow: 1.0 mL min^{-1}. The MS detector was configured with an electrospray ionization source (Micromass ZQ4000) and nitrogen was used as the

nebuliser gas. Data acquisition was performed with MassLynx software. ^{1}H-NMR spectra were recorded using Bruker Avance III spectrometer operated at 500 MHz. ^{13}C-NMR spectra were recorded using Bruker Avance operated at 126 MHz. Chemical shifts were reported in ppm on the δ scale relative to a residual solvent ($CDCl_3$: δ = 7.26 for ^{1}H-NMR and 77.16 for ^{13}C-NMR, CD_3OD: δ = 3.31 for ^{1}H-NMR and 49.00 for ^{13}C-NMR). Multiplicities are referred by the following abbreviations: s = singlet, d = doublet, t = triplet, dd = doublet doublets, ddd = double double doublet, dt = double triplet, q = quartet and m = multiplet.

Photophysical calculations. Spectroscopic data were recorded on a Synergy HT spectrophotometer (Biotek). Compounds were dissolved at the indicated concentrations and spectra were recorded at 37 °C.

In vitro characterization assays. Enzymatic assays were run in a 384-well format in PCR opaque microplates (Thermo Scientific). All experiments were performed at least in triplicate. hGzmA assays were performed in Tris buffer (0.05 M TRIS pH 8.0 containing 0.15 M NaCl and 0.01% Triton X-100). mGzmA assays were performed in Tris buffer (0.1 M TRIS, pH 8.5). hGzmB assays were performed in Tris buffer (0.05 M Tris-HCl, 0.1 M NaCl, pH 7.4). Human neutrophil elastase assays were performed in Tris buffer (0.05 M Tris, 1 M NaCl, 0.01% m/v Triton X-100, pH 8). hGzmK assays were performed in Tris buffer (0.05 M Tris, 1 M NaCl, 0.05% m/v Triton X-100, pH 7.5). MMP-2, -9 and -13 assays were performed in Tris buffer (0.05 M Tris, 0.2 M NaCl, 2 mM $CaCl_2$, pH 7.5). Caspase 4, 5, 6, 7, 8, 9 and 10 assays were performed in HEPES buffer (25 mM HEPES, 0.1% CHAPS, 10 mM DTT, pH 7.5). Human cathepsin B assays were performed in MES buffer (25 mM MES, pH 5.0). Human cathepsin L

assays were performed in MES buffer (25 mM MES, 5 mM DTT, pH 6.0). Following the manufacturer's recommended protocol, mouse cathepsin B was diluted to 10 μg mL^{-1} and incubated at r.t. for 15 min in activation buffer (25 mM MES, 5 mM DTT, pH 5.0) prior to the enzyme assays. Mouse cathepsin B assays were performed in MES buffer (25 mM MES, pH 5.0) at a concentration of 0.2 ng μL^{-1}. Proteolytic activity was determined by calculating the fold change in fluorescence signal over background signal. Unless otherwise stated, probes were utilized at a concentration of 20 μM and enzyme at 20 nM. For enzymatic kinetic assays, initial rates, V_0, against the different concentrations of compound **9** were plotted to determine the maximum rate V_{max} and the Michaelis constant K_M by nonlinear regression (GraphPad Prism).

NK-92 cell activation. NK-92 cells were grown in a MEM media supplemented with horse serum (12.5%), FBS (12.5%), L-glutamine (2 mM), folic acid (20 μM), inositol (200 μM) and 2-mercaptoethanol (100 μM) in a humidified atmosphere at 37°C with 5% CO_2. Cells were regularly passaged in T-25 cell culture flasks upon reaching 90% confluency. 100 μL of PMA (0.15 μg mL^{-1}) and 100 μL Ca^{2+} ionophore (3 μg mL^{-1}) in NK-92 cell culture media were added to 100,000 NK cells and the final volume was adjusted to 500 μL with phenol-free RPMI 1640 media. All the tubes were incubated for 2 h at 37°C with 5% CO2.

NK-92 cell inhibition and imaging. 100,000 NK-92 cells were stained with Hoechst 33342 for 20 min. The cells were then stimulated with IL-2 (2,000 U mL^{-1}) and incubated in NK-92 media with 5 μM compound **9** for 1 h with and without the inhibitor 3,4-dichloroisocoumarin (1 μM). Confocal images were acquired on Leica SP8 (Leica Microsystems) and qualitatively analysed using Fiji.

Cell co-cultures, flow cytometry and real-time imaging. HaCat cells were purchased from ATCC and grown in complete DMEM. For HaCaT cell imaging, cells were plated at 2,000 cells per well in complete DMEM and incubated with hemicyanine or compound **9** (both at 5 μM) and Hoechst 33342 at 37 ºC. Confocal images were acquired on Leica SP8 and images were analysed using Leica Application Suite X. RAW264.7 cells were purchased from ATCC and were grown in complete RPMI supplemented with 10% foetal calf serum (FCS) and antibiotics (100 U mL^{-1} penicillin and 100 μg mL^{-1} streptomycin). For RAW264.7 imaging, cells were plated at 1,000 cells per well in complete RPMI and incubated with hemicyanine or compound **9** (both at 5 μM) and Hoechst 33342 at 37 ºC. For control experiment, the RAW cells were pre-incubated with 1 μM E-64 for 1 h at 37 ºC before incubated with compound **10**. Confocal images were acquired on Leica SP8 and images were analysed using Leica Application Suite X. EL4 cells were purchased from ATCC and grown in complete RMPI supplemented with 10% heat inactivated fetal bovine serum, 1% penicillin/streptomycin, 2 mM L-glutamine and 40 μM β-mercaptoethanol. For co-culture experiments, EL4 cells were pulsed with SIINFEKL peptide to be recognized by OT-I T cells. OT-I TCR transgenic T cells were generated in vitro from cell isolates from the spleen and lymph nodes of OT-I mice. Splenocytes were pulsed with SIINFEKL and put in co-culture with cells from the lymph nodes. The TCR based recognition of SIINFEKL resulted in the expansion of antigen specific OT-I T cells. Cells were expanded for 5 days and used at day 5 for cytotoxicity experiments. EL4 cells were pulsed with SIINFEKL, 2.5 μM compound **10** and 3 μM probe H5 for 1 h at 37 ºC in complete RPMI. After incubation cells were washed three times. EL4 cells were incubated with or without OT-I T cells for 4 h 37 ºC. Samples were stained with

CD8-Percp Cy5.5 antibody, DAPI and Annexin V, and acquired on the BD Fortessa flow cytometer. Flow cytometric analysis was performed using Flowjo.

For real-time imaging, EL4 cells were seeded onto fibronectin coated chambers and allowed to settle. OT-I T cells were added to the EL4 and imaged on the Nikon A1R for 4 h. Image analysis was performed using Imaris.

Measurements of mGzmA activity in mouse sera and *ex vivo* mouse tissues. All animal work was ethically approved and conducted at the University of Zaragoza under the control of the animal welfare committee (project number: 64/17). Blood was collected by cardiac puncture after pentothal administration, and sera was extracted by centrifugation. Spleen and liver tissues were harvested and lysed for *ex vivo* analysis. Compounds **9** or **10** (20 nM) were incubated with 2.25 μL of mouse serum or spleen/liver lysates in a final volume of 45 μL for 3 h at 37 ºC. Fluorescence measurements were performed in a fluorescence spectrophotometer (λ_{exc}: 680 nm).

Chemical synthesis and characterization

Solid-phase peptide synthesis.

Resin loading. Fmoc-AA-OH (1 eq) were attached to the resin with DIPEA (3 eq) in DCM at r.t. for 10 min and then DIPEA (7 eq) for 40 min. The remaining 2-chlorotrityl groups were capped by addition of MeOH (0.8 mL g^{-1} resin) for 10 min. After that, the resin was filtered off and washed with DCM (5 × 1 min), DMF (5 × 1 min). The loading of the resin was determined by titration of the Fmoc group using UV-Vis measurements.

Amino acid couplings. After the Fmoc groups were removed, resins were washed with DMF (5 × 1 min), DCM (5 × 1 min) and DMF (5 × 1 min). A solution of the

appropriate Fmoc-AA-OH (4 eq), COMU (4 eq), Oxyma (4 eq) and DIPEA (8 eq) was stirred for 1 min in DMF before addition to the resin previously swollen in DMF. The mixture was then stirred for 1 h at r.t. The solution was filtered off and the resins were washed with DMF (5 × 1 min) and DCM (5 × 1 min). The completion of the couplings was monitored by the Kaiser test.

N-terminal acetylation. After removal of the last Fmoc group, resins were washed with DMF (5 × 1 min), DCM (5 × 1 min), DMF (5 × 1 min) and then treated with a Ac_2O (10 eq) and DIPEA (10 eq) in DMF. Afterwards, the resins were filtered off and washed with DCM (5 × 1 min) and DMF (5 × 1 min).

Cleavage. Resin-bound peptides were treated with 2% TFA in DCM (5 × 1 min) and washed with DCM. The combined filtered mixtures were poured over DCM and evaporated under reduced pressure. The peptides were isolated by precipitation in ice-cold Et_2O generating white solids (HPLC purities > 95%).

Protecting group removal in solution. The peptides were treated with a mixture of 95% TFA, 2.5% TIPS and 2.5% DCM for 1 h and evaporated under reduced pressure. The peptides were isolated by precipitation in ice-cold Et_2O generating white solids.

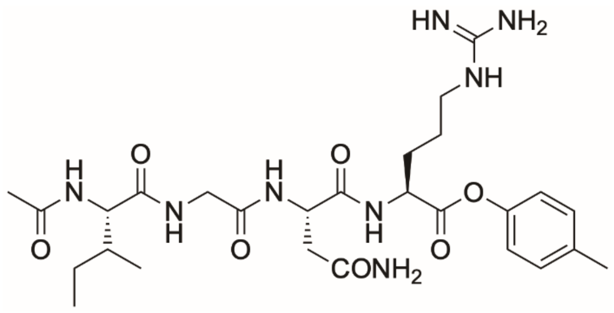

Compound 2. Ac-Ile-Gly-Asn-Arg-OH (**1**) (20 mg, 0.04 mmol), p-cresol (8.6 mg, 0.08 mmol), EDC HCl (11.4 mg, 0.06 mmol) and DMAP (0.5 mg, 0.004 mmol) were dissolved in anhydrous DMF (0.6 mL) and the reaction mixture was stirred overnight.

Purification was performed via reverse-phase preparative HPLC to yield a white powder (1 mg).

t_R = 4.88 min (99% purity). HRMS (ESI+): m/z calc. for $C_{27}H_{41}N_8O_7$: 589.3093; found: 589.3079.

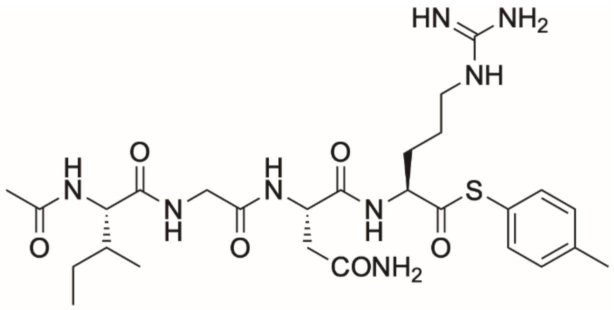

Compound 3. Ac-Ile-Gly-Asn-Arg-OH (60 mg, 0.12 mmol), PyBOP (315 mg, 0.6 mmol) and 4-methylbenzenethiol (448 mg, 36 mmol) were dissolved in anhydrous DMF under N_2 atmosphere and the reaction mixture was stirred for 2 h. Purification was performed by reverse-phase preparative HPLC to yield a white powder (2.5 mg). t_R = 5.03 min (90% purity). HRMS (ESI+): m/z calc. for $C_{27}H_{43}N_8O_6S_1$: 607.3021; found: 607.3013.

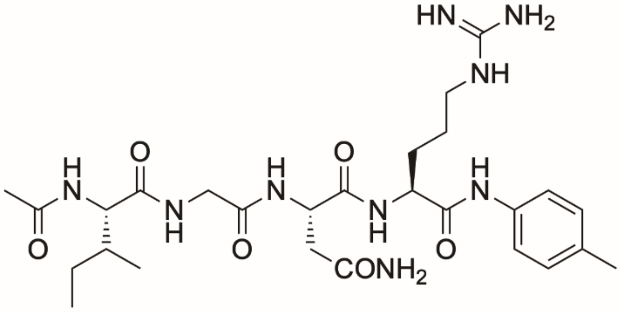

Compound 4. Ac-Ile-Gly-Asn-Arg-OH (50 mg, 0.1 mmol), p-toluidine (55 mg, 0.5 mmol) and EEDQ (138 mg, 0.5 mmol) were added into a round bottom flask under N_2 atmosphere. Anhydrous DMF (4 mL) was added, and the mixture was stirred at r.t.

overnight. Purification was performed using reverse-phase preparative HPLC to render a white powder (15.5 mg, 26 %).

t_R = 4.75 min (99% purity). HRMS (ESI+): m/z calc. for $C_{27}H_{44}N_9O_6$: 590.3409; found: 590.3389.

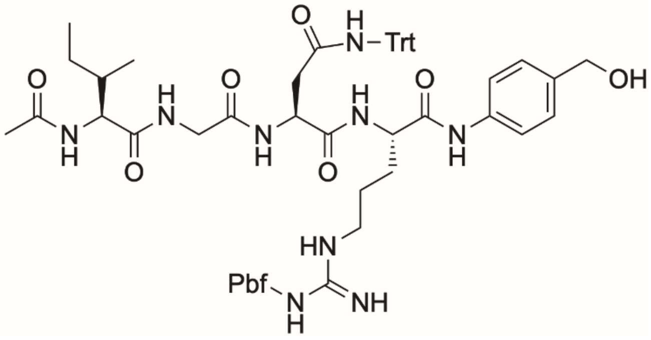

Compound 6. Ac-Ile-Gly-Asn(Trt)-Arg(Pbf)-OH (300 mg, 0.3 mmol), 4-aminobenzyl alcohol (189 mg, 1.5 mmol) and EEDQ (418 mg, 1.5 mmol) were added into a round bottom flask under N_2 atmosphere. Anhydrous DMF (6 mL) was added, and the mixture was stirred overnight. Purification was performed using reverse-phase preparative HPLC to yield a white powder (140 mg, 42%).

t_R = 8.12 min (99% purity). HRMS (ESI+): m/z calc. for $C_{59}H_{74}N_9O_{10}S_1$: 1100.5274; found: 1100.5249.

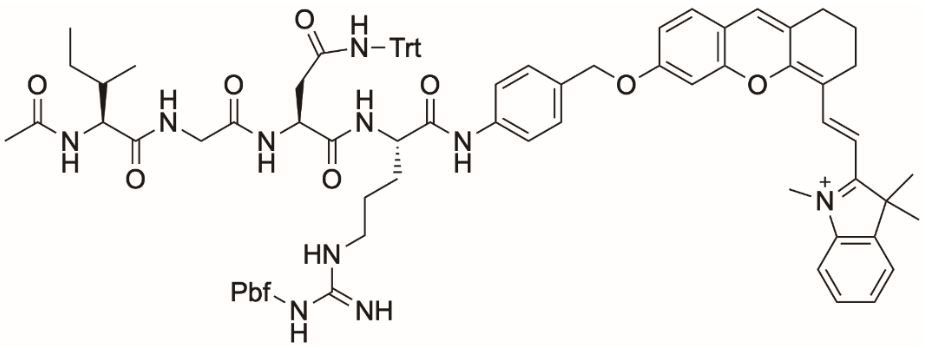

Compound 8. Compound **6** (70 mg, 0.064 mmol) was dissolved in anhydrous THF under N_2 atmosphere and was cooled to 0 °C. PBr_3 (9 µL, 0.0954 mmol) was then

added and the reaction mixture was stirred for 3 h at 0 °C. THF was removed, and DCM was added. The mixture was washed with saturated $NaHCO_3$ twice and the organic layers were collected, dried over $MgSO_4$, filtered and evaporated to yield compound **7**, which was used without further purification. Anhydrous ACN (3 mL) was then added to compound **7** before the hemicyanine fluorophore (19.5 mg, 0.05 mmol) and DIPEA (26 µL, 0.15 mmol) were added, and the reaction mixture was stirred overnight. Purification was performed using reverse-phase preparative HPLC to yield a deep blue powder (4 mg).

t_R = 8.25 min (99% purity). MS (ESI+): m/z calc. for $C_{85}H_{97}N_{10}O_{11}S_1$: 1465.7; found: 1466.9.

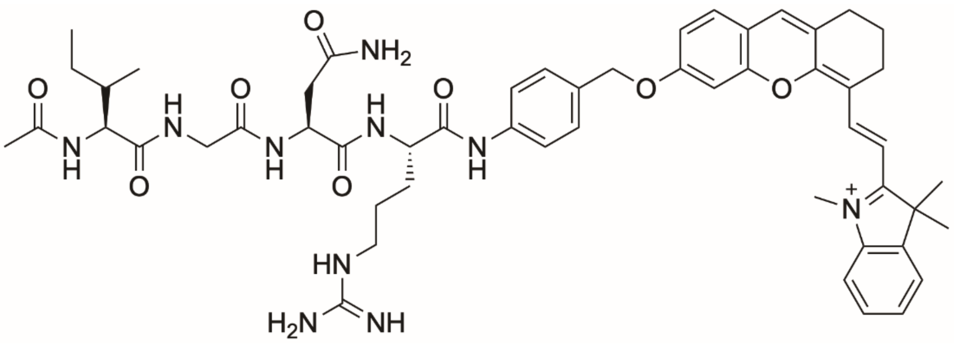

Compound 9. Compound **8** was treated with 2 mL of a solution consisting of 95% TFA, 2.5% TIPS and 2.5% DCM for 15 min. The TFA was evaporated, and the crude mixture was redissolved in MeOH. Purification was performed using reverse-phase preparative HPLC to yield a blue powder (1.2 mg).

t_R = 6.02 min (95% purity). HRMS (ESI+): m/z calc. for $C_{53}H_{67}N_{10}O_8$: 971.5138; found: 971.5155.

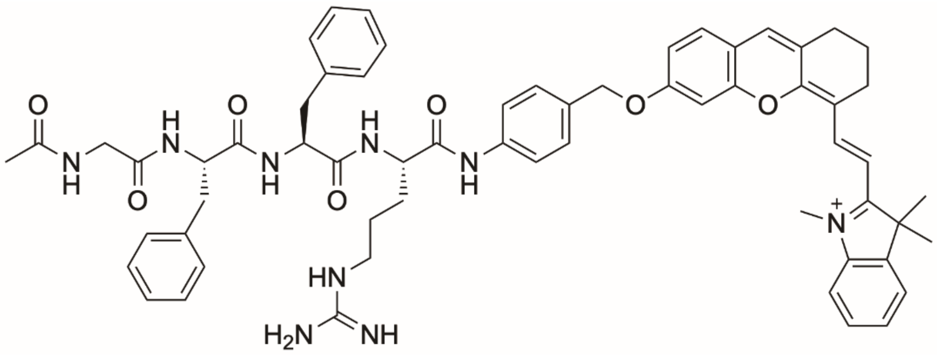

Compound 10. Compound **15** (63 mg, 0.068 mmol) was dissolved in anhydrous THF (40 mL) under N_2 atmosphere and cooled to 0 °C. PBr_3 (9.9 μL, 0.1 mmol) was added, and the reaction mixture was stirred for 3 h at 0 °C. THF was removed, and DCM was added. The mixture was washed with saturated $NaHCO_3$ twice and the organic layers were collected, dried over $MgSO_4$, filtered off and evaporated to yield off-white solids. Anhydrous ACN was then added to the off-white solids before hemicyanine (26 mg, 0.068 mmol) and DIPEA (22 μL, 0.136 mmol) were added. The reaction mixture was stirred overnight. The solvent was evaporated under vacuo and was subsequently treated with 5 mL solution of TFA:DCM (95:5) solution for 15 min. Purification was performed using reverse-phase preparative HPLC and lyophilised to render a blue solid (0.5 mg).

t_R = 3.60 min (99% purity). HRMS (ESI+): m/z calc. for $C_{61}H_{68}N_9O_7$: 1038.5236; found: 1038.5223.

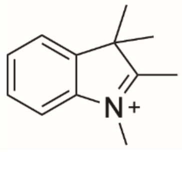

Compound 11. To a solution of 2,3,3-trimethylindolenine (1.63 mL, 10 mmol) in acetonitrile (20 mL), methyl iodide (943 μL, 15 mmol) was added, and the reaction mixture was heated at reflux for 12 h. The resulting solid was filtered using a Buchner

funnel and washed with Et_2O. The light reddish solids were dried overnight under vacuum and used without further purification.

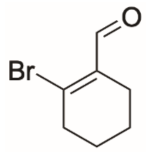

Compound 12. To a solution of DMF (2.24 mL, 30 mmol) in $CHCl_3$ (10 mL) at 0°C was slowly added PBr_3 (2.48 mL, 30 mmol) and stirred for 45 min. Cyclohexanone (1 mL, 10 mmol) was then added and the reaction mixture was stirred for 16 h at r.t. The red solution was poured onto ice, and solid $NaHCO_3$ was slowly added until the solution reached pH 7. The layers were separated, and the aqueous layer was extracted with DCM (×3). All organic layers were collected and dried over $MgSO_4$, filtered and concentrated under vacuo to yield a yellow liquid. The yellow liquid was used without further purification.

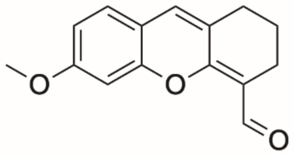

Compound 13. Compound **12** (3.54 g, 20 mmol) was stirred in DMF and 2-hydroxy-4-methoxybenzaldehyde (2.14 g, 14 mmol) and Cs_2CO_3 (13.68 g, 42 mmol) were added and stirred for 16 h at 25 °C. The green solution was filtered through a pad of silica gel on a Buchner funnel. The filtrate was concentrated, dissolved in DCM and washed with water (×2). The organic layers were combined, dried over $MgSO_4$, filtered off and concentrated under vacuo. Purification via silica gel chromatography was performed using eluent (0 to 10 % EA in Hexane) to yield a yellow solid (369 mg).

^{1}H NMR (500 MHz, CDCl3) δ 10.32 (s, 1H), 6.69 – 6.63 (m, 5H), 3.84 (s, 5H), 2.57 (ddt, J = 7.3, 4.9, 1.1 Hz, 3H), 2.45 (t, J = 6.0 Hz, 3H), 1.76 – 1.67 (m, 3H).

^{13}C NMR (126 MHz, CDCl3) δ 187.5, 161.3, 160. 7, 153.3, 127.3, 126.7, 126.5, 114.6, 112.5, 110. 8, 100.4, 55.6, 29.8, 21.4, 20.3.

t_R = 8.15 min (99% purity). HRMS (ESI+): m/z calc. for $C_{15}H_{15}O_3$: 243.1016; found: 243.1019.

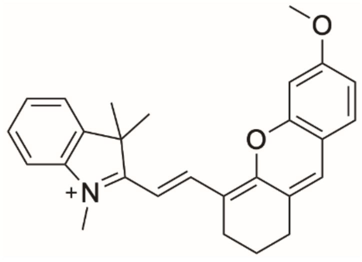

Compound 14. Compound **13** (300 mg, 1 mmol) was added to a round bottom flask with Ac_2O (10 mL), and K_2CO_3 (342 mg, 2.5 mmol) was added. Compound **11** (260 mg, 1.5 mmol) was then added and stirred for 16 h at 80 °C. The blue solution was then concentrated under vacuo and the residue was redissolved in DCM before washing with water (×3). The organic layers were collected and dried with $MgSO_4$, filtered and evaporated under vacuo. Purification via silica gel (0 to 20% MeOH in DCM) rendered a blue solid (283 mg).

^{1}H NMR (500 MHz, CDCl3) δ 8.65 (d, J = 14.9 Hz, 1H), 7.52 – 7.44 (m, 2H), 7.44 – 7.31 (m, 3H), 7.20 (s, 1H), 6.97 – 6.85 (m, 2H), 6.61 (d, J = 14.9 Hz, 1H), 4.10 (s, 3H), 3.97 (s, 3H), 2.86 (t, J = 6.1 Hz, 2H), 2.74 (t, J = 1.4 Hz, 2H), 1.96 (q, J = 6.2 Hz, 2H), 1.81 (s, 6H).

^{13}C NMR (126 MHz, CDCl3) δ 178.0, 163.4, 161.9, 154.9, 146.1, 142.6, 141.9, 133.7, 129.6, 129.0, 127.9, 127.6, 122.6, 116.1, 115.8, 113.4, 113.1, 105.4, 101.3, 56.8, 50.9, 35.4, 29.7, 28.6 (2C), 25.3, 20.7.

t_R = 7.03 min (99% purity). HRMS (ESI+): m/z calc. for $C_{27}H_{28}N_1O_2$: 398.2115; found: 398.2126.

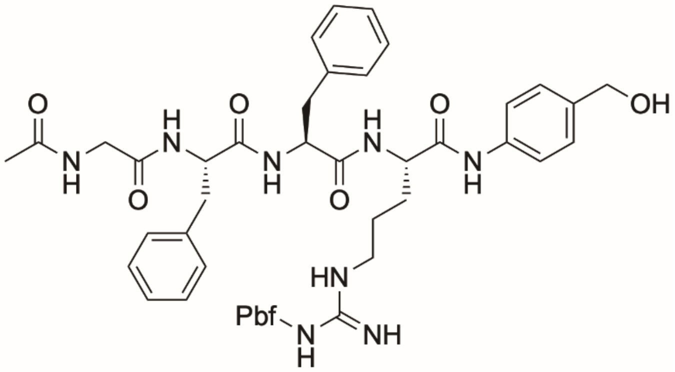

Compound 15. Ac-Gly-Phe-Phe-Arg(Pbf)-OH (230 mg, 0.28 mmol), 4-aminobenzyl alcohol (176 mg, 1.4 mmol) and EEDQ (389 mg, 1.4 mmol) were added into a round bottom flask under N_2 atmosphere. Anhydrous DMF (10 mL) was added, and the mixture was stirred overnight. Purification was performed using reverse-phase preparative HPLC and lyophilised to render a white powder (126 mg, 48%).
t_R = 7.12 min (99% purity). HRMS (ESI+): m/z calc. for $C_{48}H_{61}N_8O_9S_1$: 925.4277; found: 925.4272.

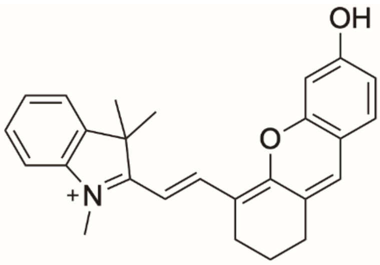

Hemicyanine. Compound **14** (0.283 g, 0.502 mmol) was dissolved in DCM (15 mL) under N_2 atmosphere and cooled to 0 °C. BBr_3 in DCM (1 M, 25 mL, 25 mmol) was added and stirred at r.t. overnight. The reaction was quenched by adding saturated $NaHCO_3$ at 0 °C, and the organic layers were separated from the aqueous layers. The aqueous layers were washed twice with DCM and all organic solvents were collected,

dried over $MgSO_4$, filtered off and evaporated. Purification via silica gel (0 to 20% MeOH in DCM) rendered a blue solid (148 mg).

^{1}H NMR (500 MHz, MeOD) δ 8.74 (d, J = 14.8 Hz, 1H), 7.65 – 7.60 (m, 1H), 7.55 – 7.46 (m, 2H), 7.46 – 7.38 (m, 3H), 6.87 – 6.81 (m, 2H), 6.43 (d, J = 14.8 Hz, 1H), 3.81 (s, 3H), 2.79 (t, J = 6.0 Hz, 2H), 2.73 (t, J = 6.2 Hz, 2H), 1.94 (t, J = 6.1 Hz, 2H), 1.81 (s, 6H).

^{13}C NMR (126 MHz, MeOD) δ 178.8, 163.7, 156.6, 146.4, 144.1, 143.2, 136.6, 130.6, 130.3, 128.0, 127.3, 123.7, 116.5, 116.5, 115.7, 113.4, 103.9, 103.1, 51.7, 32.6, 30.1, 28.4 (2C), 25.2, 21.9.

t_R = 6.52 min (99% purity). HRMS (ESI+): m/z calc. for $C_{26}H_{26}N_1O_2$: 384.1958; found: 384.1970.

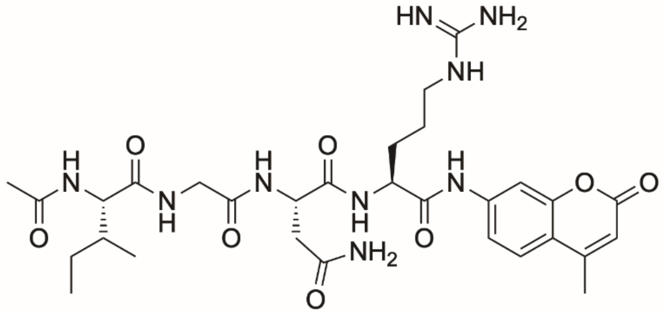

Ac-IGNR-AMC. Ac-Ile-Gly-Asn(Trt)-Arg(Pbf)-OH (100 mg, 0.1 mmol) and 7-amino-4-methylcoumarin (21.6 mg, 0.12 mmol) and HATU (76 mg, 0.2 mmol) were weighed into a round bottom flask. Under N_2 atmosphere, anhydrous DMF (5 mL) and DIPEA (0.053 mL, 0.3 mmol) were added, and the reaction mixture was stirred for 24 h. The DMF was then removed under reduced pressure. Next, a 2 mL solution of TFA:TIPS:DCM (95:2.5:2.5) was added to the mixture and stirred for 15 min. The solvent was evaporated under reduced pressure and the crude mixture was purified by preparative HPLC to yield a white powder (2 mg).

t_R = 4.73 min (99% purity). HRMS (ESI+): m/z calc. for $C_{30}H_{44}N_9O_8$: 658.3307; found: 658.3322.

NMR Spectra

Compound 13

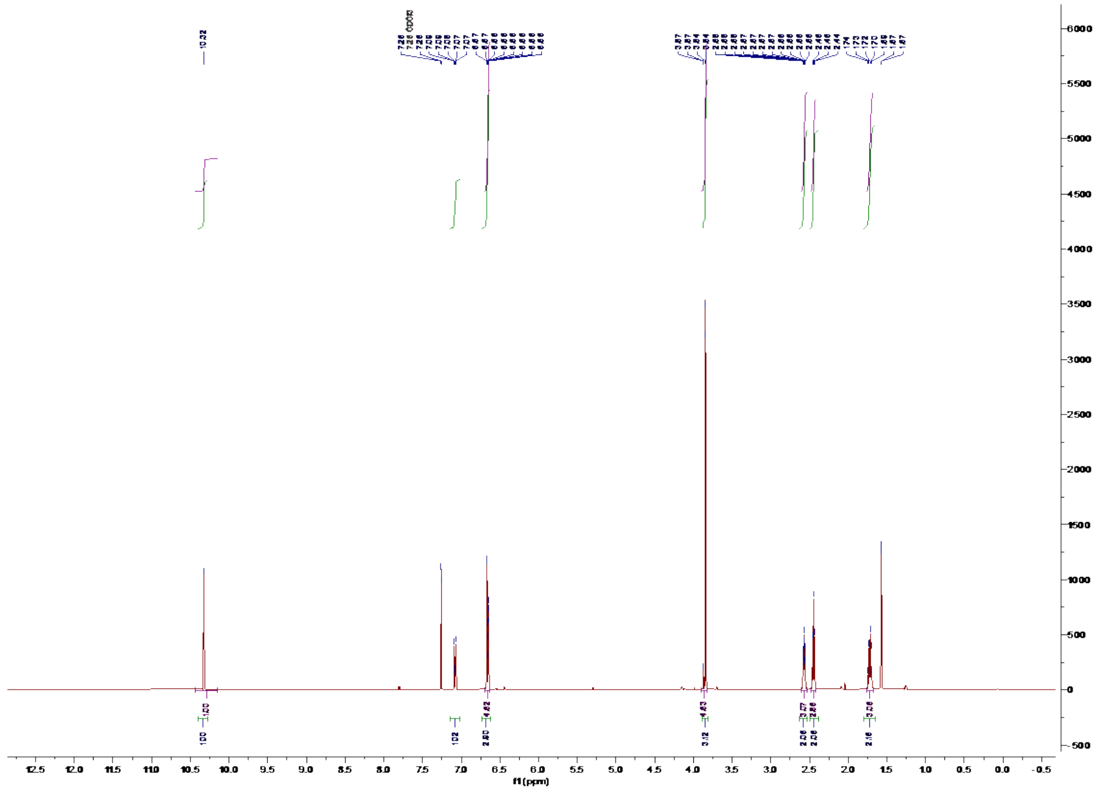

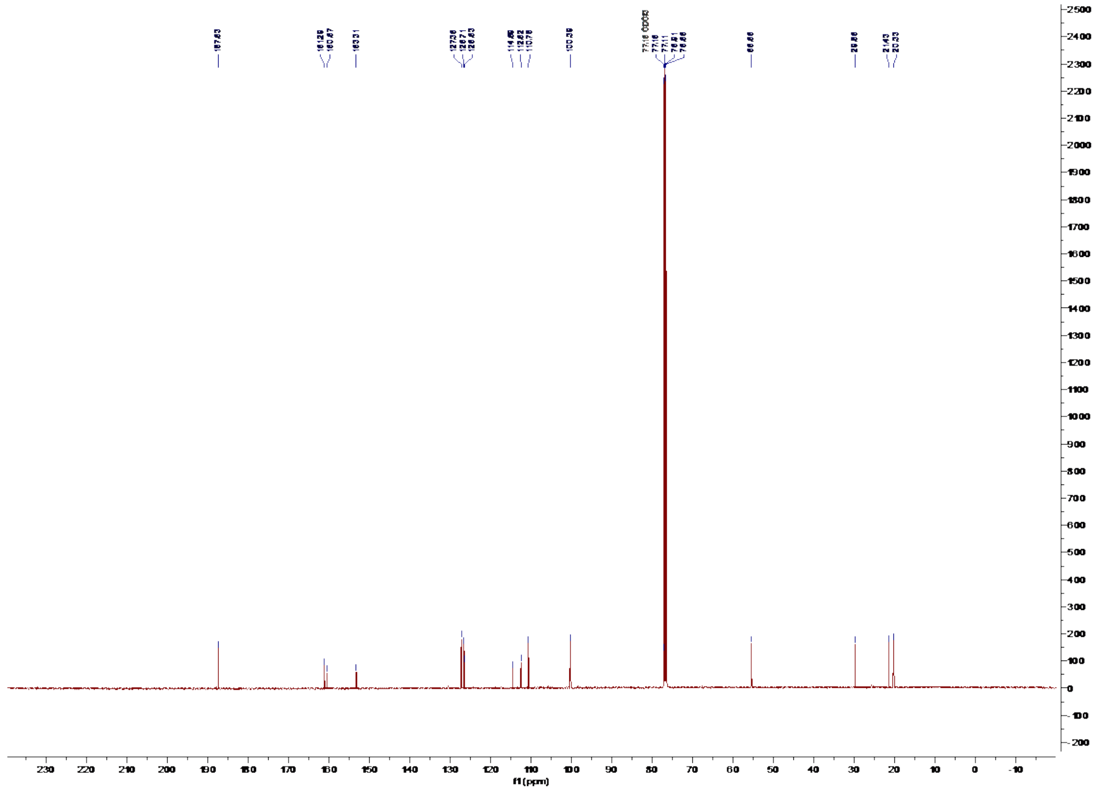

Compound 14

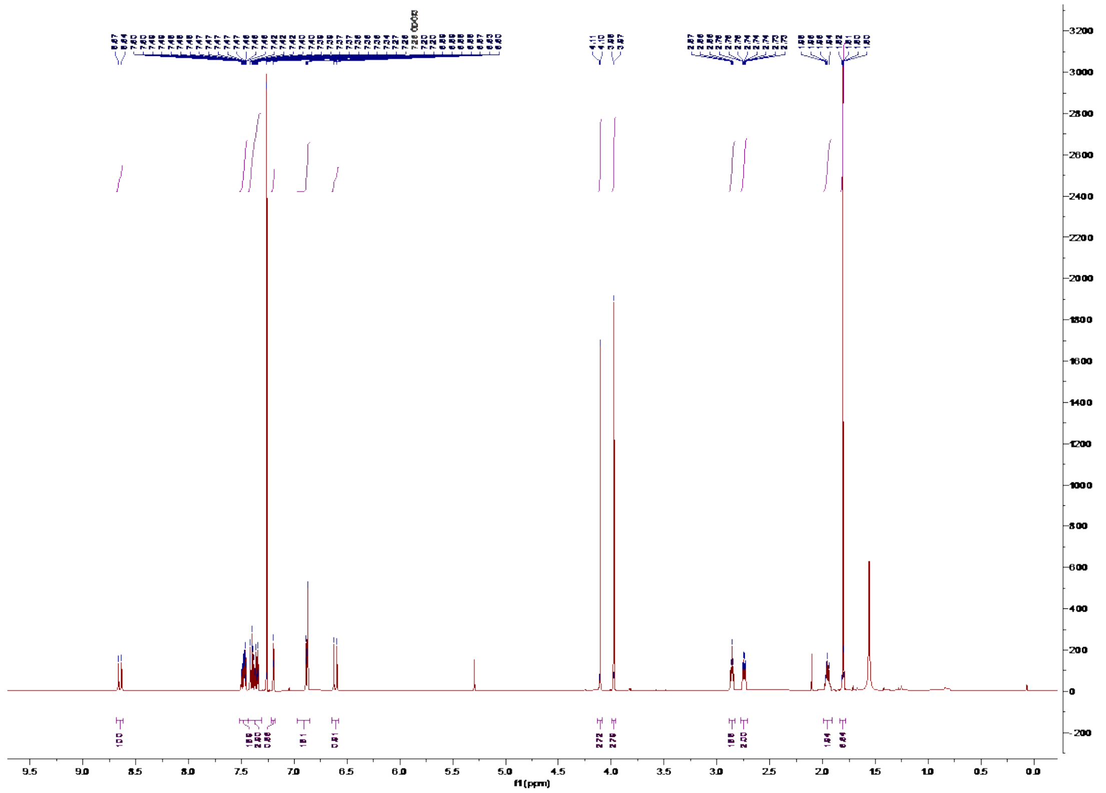

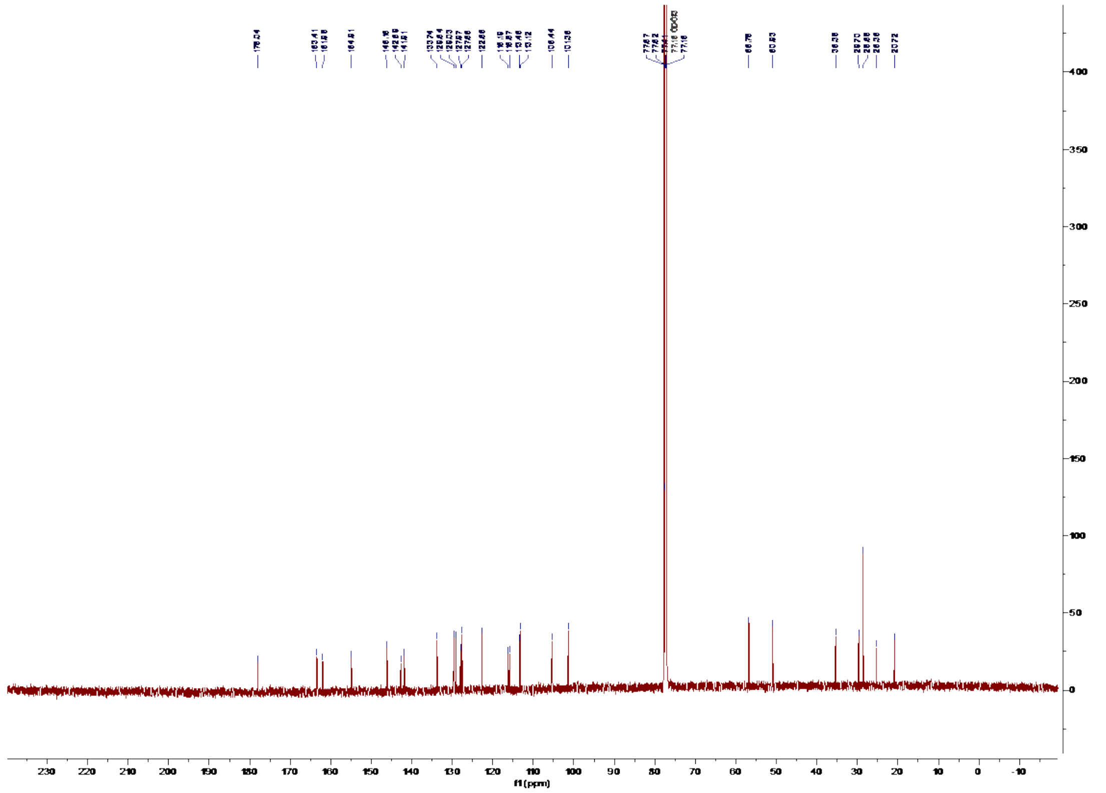

Hemicyanine

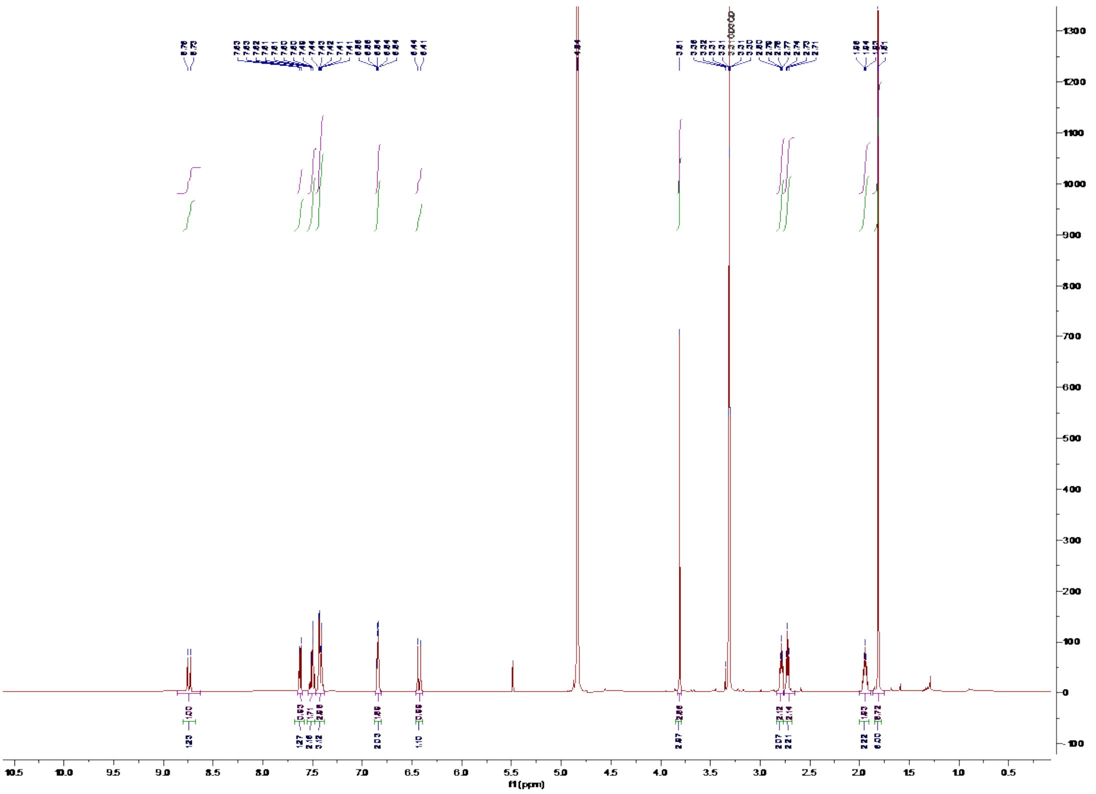

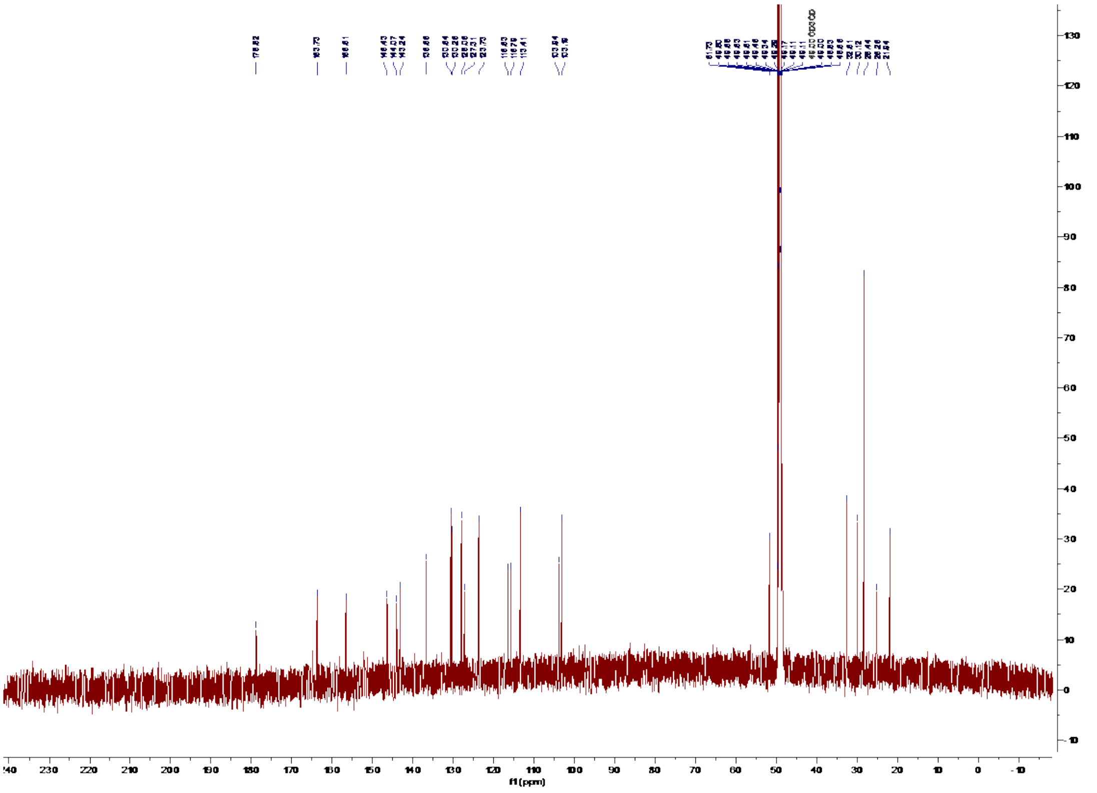

Supplementary References

[1] J. I. Scott, L. Mendive-Tapia, D. Gordon, N. D. Barth, E. J. Thompson, Z. Cheng, D. Taggart, T. Kitamura, A. Bravo-Blas, E. W. Roberts, J. Juarez-Jimenez, J. Michel, B. Piet, I. J. de Vries, M. Verdoes, J. Dawson, N. O. Carragher, R. A. O'Connor, A. R. Akram, M. Frame, A. Serrels, M. Vendrell, *Nat Commun* **2022**, *13*, 2366.

[2] J. A. Richard, *Org Biomol Chem* **2015**, *13*, 8169-8172.
